# OTUD4-ZMYND8-DDX3X Axis Drives Immunosuppressive Microenvironment in Spinal Metastases of Triple-Negative Breast Cancer

**DOI:** 10.1016/j.neo.2025.101259

**Published:** 2025-11-25

**Authors:** Bing Liang, Annan Hu, Hongwei Lu, Hao Zhou, Qing Chen, Chao Jia, Jinjin Wang, Libo Jiang, Wei Hong, Jian Zhou, Jian Dong

**Affiliations:** a Department of Orthopaedic Surgery, Zhongshan Hospital, Fudan University, Shanghai 200032, China; b Department of Orthopaedic Surgery, Shanghai Geriatric Medical Center, Shanghai 201104, China; c Department of Orthopaedic Surgery, Shanghai Xuhui Central Hospital, Zhongshan-Xuhui Hospital, Fudan University, Shanghai 200031, China; d Department of Geriatrics and Gerontology, Huadong Hospital, Fudan University, Shanghai 200040, China; e State Key Laboratory of Molecular Engineering of Polymers, Fudan University, Shanghai 200438, China; f Department of Orthopaedic Surgery, Zhongshan Hospital Wusong Branch, Fudan University, Shanghai 200940, China

**Keywords:** Spinal metastasis, Triple-negative breast cancer, ZMYND8, OTUD4, DDX3X

## Abstract

Triple-negative breast cancer (TNBC) exhibits a high propensity for spinal metastasis, leading to severe morbidity and limited therapeutic responses. However, the molecular mechanisms driving spinal colonization remain poorly defined. Here, we identify the epigenetic reader ZMYND8 as a key mediator of TNBC spinal metastasis. ZMYND8 is significantly upregulated in spinal metastatic lesions and correlates with adverse patient outcomes. Transcriptomic profiling reveals that spinal metastases display profoundly immunosuppressive microenvironments, with elevated M2 macrophage infiltration positively associated with ZMYND8 expression. Mechanistically, ZMYND8 functions as a scaffold protein that promotes assembly of the DDX3X–CK1ε complex, thereby activating WNT/β-catenin signaling and promoting spinal metastasis. Furthermore, we identify OTUD4 as a bona fide deubiquitinase that directly interacts with and stabilizes ZMYND8, thereby enhancing TNBC cell migration, invasion, and spinal colonization. The resulting OTUD4–ZMYND8–DDX3X signaling axis drives canonical WNT/β-catenin signaling, upregulates CSF1 expression and promotes M2 polarization of macrophages, collectively fostering invasive behavior and establishing an immunosuppressive niche conducive to spinal metastasis. Collectively, these findings establish the OTUD4–ZMYND8–DDX3X axis as a pivotal regulator of spinal metastasis in TNBC and highlight its potential as a therapeutic target for inhibiting metastatic progression.

## Highlights

• The OTUD4-ZMYND8-DDX3X axis orchestrates spinal metastasis in triple-negative breast cancer.
• ZMYND8 functions as a molecular scaffold for the DDX3X-CK1ε complex to activate Wnt/β-catenin.
• The deubiquitinase OTUD4 directly binds to and stabilizes ZMYND8.
• ZMYND8 upregulation promotes an immunosuppressive niche with M2 macrophage polarization.
• This study unveils a novel signaling axis as a promising therapeutic vulnerability.

## Introduction

Breast cancer is the most common malignancy globally and the leading cause of cancer-related morbidity among women [1,2]. Although advances in early detection and treatment have improved outcomes, metastatic disease remains the principal cause of death, with bone metastasis ranking among the most frequent and severe complications [[3], [4], [5], [6]]. Triple-negative breast cancer (TNBC) represents a particularly aggressive subtype of breast carcinoma, for which limited targeted therapeutic options are currently available [7,8]. >70 % of patients with advanced TNBC develop bone metastases, frequently resulting in skeletal-related events including pathological fractures, severe pain, and functional impairment [5,9,10]. The vertebral column, with its rich vascularization and unique microenvironment, is a preferred site for metastatic colonization [11,12]. Spinal metastases significantly reduce quality of life and can cause serious neurologic sequelae such as spinal cord compression and paralysis, further complicating clinical management and worsening prognosis. Recent investigations have emphasized the dynamic interplay between breast cancer cells and the immune microenvironment, revealing a key role for immune evasion in metastatic progression [[13], [14], [15]]. Additionally, co-evolution of tumor and immune cells supports metastatic dissemination to distant sites including bone, lung, liver, and brain [12,16]. Understanding the mechanisms driving spinal metastasis is therefore critical for developing strategies to inhibit systemic spread and improve patient survival. Despite progress, the molecular and metabolic mechanisms underlying spinal metastasis in TNBC remain inadequately characterized, and targeted therapeutic modalities for this devastating condition are still emerging.

ZMYND8 (Zinc finger MYND-type containing 8), also referred to as RACK7 or PRKCBP1, is a multidomain epigenetic reader that recognizes specific histone modifications and recruits transcriptional complexes to regulate gene expression [[17], [18], [19], [20]]. Functionally, ZMYND8 exhibits context-dependent roles in oncogenesis: it activates oncogenic transcription by recruiting HIF-1α, BRD4, and P-TEFb to augment hypoxia response, lipid metabolism, and cellular proliferation [[21], [22], [23], [24], [25]], yet also represses tumor-suppressive pathways through associations with KDM5C, NuRD, and EZH2 complexes [[26], [27], [28]]. Notably, ZMYND8 is overexpressed in breast cancer, where it promotes metastasis through dysregulation of HIF signaling, the cGAS-STING pathway, and cholesterol metabolism [21,29,30]. This pro-metastatic function extends to other malignancies, including glioblastoma, renal carcinoma, and bladder cancer, establishing ZMYND8 as a conserved oncogenic driver [22,24,28,[31], [32], [33]]. Despite its established oncogenic role, the post-translational mechanisms controlling ZMYND8 protein stability are poorly defined. FBXW7-mediated ubiquitination targets ZMYND8 for proteasomal degradation [33], whereas a recent study identified USP7 as a deubiquitinase that stabilizes ZMYND8 and thereby promotes breast cancer cell migration and invasion[34]. Collectively, these findings position ZMYND8 within a dynamic regulatory network of E3 ligases and deubiquitinases (DUBs) and imply that additional DUBs may regulate ZMYND8 in a context-dependent fashion influenced by cellular state and the tumor microenvironment, particularly in TNBC and spinal metastatic niches.

OTUD4 (OTU domain-containing protein 4) is a member of the ovarian tumor deubiquitinase family that catalyzes the removal of ubiquitin chains from substrate proteins, thereby counteracting proteasomal degradation and enhancing protein stability [35,36]. This enzyme preferentially cleaves K48-linked polyubiquitin chains via its catalytic triad (Ser-His-Asp) and serves as a key regulator of diverse cellular processes, including antiviral immunity, DNA repair, and metabolic reprogramming [[35], [36], [37]]. Accumulating evidence indicates that OTUD4 plays context-dependent roles in tumorigenesis: it facilitates metastasis in TNBC and melanoma by stabilizing Snail1 [38,39], yet suppresses tumor progression in non-small cell lung cancer and hepatocellular carcinoma through distinct molecular targets [40,41]. Of clinical relevance, elevated OTUD4 expression is associated with poorer outcomes in breast cancer and facilitates TGF-β signaling—a pathway critically involved in epithelial–mesenchymal transition (EMT) and metastatic dissemination [39,42]. However, the functional interplay between OTUD4 and epigenetic regulators during breast cancer metastasis remains inadequately explored. Although OTUD4 is known to stabilize transcription factors such as Snail1 and signaling adaptors like MAVS, its potential interaction with chromatin-reading proteins—including ZMYND8, which transcriptionally activates metastasis-promoting genes such as ZEB1 and VEGFA—has not been investigated [37,38]. Notably, aberrant accumulation of ZMYND8 has been observed in breast tumors, yet the mechanisms enabling its stabilization in malignancy are poorly defined. In our preliminary ZMYND8 interactome analysis, OTUD4 emerged as a high-confidence binding partner in TNBC models, whereas USP7 was not detected in human MDA-MB-231 cells and was recovered at much lower abundance than OTUD4 in murine 4T1 cells. Given that ZMYND8 is subject to FBXW7-mediated ubiquitination but frequently escapes degradation in metastatic contexts, we hypothesized that OTUD4 may function as a context-dependent deubiquitinase that stabilizes ZMYND8 and thereby promotes TNBC progression.

In this study, we sought to define the biological function of ZMYND8 in triple-negative breast cancer spinal metastasis (TNBC-SM) and to elucidate the molecular mechanisms underlying its aberrant accumulation. Our initial assessment revealed that ZMYND8 is significantly upregulated in human TNBC-SM lesions, and its high expression correlates with an immunosuppressive tumor microenvironment, characterized by M2 macrophage infiltration, and poor patient survival. Mechanistically, we identified a critical scaffolding function for ZMYND8 in facilitating the assembly of a productive complex between DDX3X and CK1ε, leading to activation of the WNT/β-catenin signaling pathway. Furthermore, employing integrated proteomic and biochemical approaches, we identified OTUD4 as a bona fide deubiquitinase that directly interacts with ZMYND8, removes its ubiquitin chains, and enhances its protein stability, thereby potentiating TNBC cell migration, invasion, and spinal metastasis *in vivo*. Collectively, our findings establish the OTUD4–ZMYND8–DDX3X axis as a pivotal mechanism driving spinal metastasis in TNBC, revealing new avenues for therapeutic intervention. Our findings provide a comprehensive perspective on biomarker applications, pathogenic mechanisms, and targeted interventions involving ZMYND8 in TNBC.

## Materials and methods

### Study approval

All animal experiments were conducted in accordance with protocols approved by the Animal Care and Use Committee of Zhongshan Hospital, Fudan University (Shanghai, China) (Approval No 2024-038). The use of clinical specimens was authorized by the Medical Ethics Committee of Zhongshan Hospital, Fudan University (Shanghai, China) (Approval No B2023-296).

### Clinical specimens

Human triple-negative breast cancer (TNBC) and TNBC spinal metastasis (TNBC-SM) tissue specimens were acquired from Zhongshan Hospital, Fudan University (Shanghai, China). All participants provided written informed consent before sample collection.

### Cell lines and cell culture

The human breast cancer cell line MDA-MB-231 (RRID:CVCL_0062), the monocytic cell line THP-1 (RRID:CVCL_0006), the human embryonic kidney cell line HEK-293T (RRID:CVCL_0063), and the murine breast cancer cell line 4T1 (RRID:CVCL_0125) were acquired from the Chinese Academy of Sciences Cell Bank (Shanghai, China). The immortalized bone marrow-derived macrophage (iBMDM) cell line was a gift from Dr. Feng Shao (National Institute of Biological Science, Beijing, China). This specific iBMDM subline, widely used in the field of innate immunity and validated in previous publications, does not have a unique RRID assigned in public databases (e.g., Cellosaurus) at the time of submission. Its identity and functionality were confirmed by relevant morphological and immunological assays (e.g., response to LPS/IFN-γ stimulation). MDA-MB-231, HEK-293T, and iBMDM cells were cultured in DMEM containing 10 % fetal bovine serum (FBS) and 1 % penicillin–streptomycin. 4T1 cells were maintained in RPMI 1640 medium supplemented with 10 % FBS and 1 % penicillin–streptomycin. THP-1 cells were grown in RPMI 1640 medium with 10 % FBS, 2 mM l-glutamine, 0.05 mM β-mercaptoethanol, and 1 % penicillin–streptomycin; macrophage differentiation was induced by treatment with 320 nM phorbol 12-myristate 13-acetate (PMA) for 24 hours [43]. All cells were cultured at 37 °C in a humidified atmosphere of 5 % CO₂. All cell lines were routinely tested and confirmed to be free of Mycoplasma contamination using the BeyoColor™ Mycoplasma Detection Kit (Beyotime, C0305S). Cell line authentication was performed by short tandem repeat (STR) profiling.

### Lentiviral transduction, siRNA transfection, and mutant construction

To generate stable overexpression cell lines, the coding sequences (CDSs) of target genes were subcloned into the pLenti-GIII-CMV-CBH-GFP-2A-Puro lentiviral vector. For the establishment of ZMYND8-knockout cell lines, three sgRNAs targeting ZMYND8 were designed and cloned into the pLenti-U6-sgRNA-SFFV-Cas9-2A-Puro All-in-One lentivector. Lentiviruses were produced using a second-generation packaging system (abm, China) and used to transduce target cells. Stably transduced cells were selected under continuous puromycin treatment, and single clones were isolated, expanded, and validated by Sanger sequencing and immunoblotting. siRNA targeting OTUD4 was synthesized by Genechem Biotech (Shanghai, China) and transfected transiently into cells at approximately 60 % confluency using Lipofectamine 3000. Expression plasmids encoding His-ZMYND8, Flag-OTUD4, HA-ubiquitin (Ub), the human OTUD4 catalytic-inactive variants C45S and C45S/H148A, as well as the human ZMYND8 lysine-to-arginine substitution mutants K294R, K356R, and K508R and the corresponding mouse ZMYND8 mutants K318R, K380R, and K532R, were obtained from Genechem Biotech (Shanghai, China). All transfections were performed with Lipofectamine 3000 according to the manufacturer’s protocol.

### RNA extraction and quantitative real-time PCR (qRT-PCR) analysis

Total cellular RNA was extracted using TRIzol reagent (Invitrogen, USA) and quantified with a Denovix spectrophotometer (DeNovix Inc., USA). Complementary DNA (cDNA) was synthesized from the isolated total RNA using a cDNA synthesis kit (TaKaRa, China). Quantitative real-time PCR was subsequently performed with qPCR SYBR Green Master Mix (Yeasen, China) on an ABI QuantStudio 7 Pro system (Thermo Fisher, USA) in accordance with the manufacturer’s protocols. Relative mRNA expression levels were calculated using the 2^(−ΔΔCT) method, with normalization to *GAPDH* as an internal control. All assays were conducted in at least three independent replicates. The sequences of the qRT-PCR primers employed in this study are provided in Table S1 (Supporting Information).

### CCK-8 assay

Cell viability was assessed using the CCK-8 assay (Enhanced Cell Counting Kit-8, Beyotime, Shanghai, China) in accordance with the manufacturer's instructions. Briefly, breast cancer cells were seeded at equal densities in 96-well plates at a volume of 100 μL per well. Every 24 hours, 10 μL of CCK-8 solution was added to each well, followed by incubation at 37 °C for 2 hours in the dark. Absorbance was measured at 450 nm using a SpectraMax iD3 Multifunctional Microplate Reader (Molecular Devices, USA). The data obtained were normalized and presented as a line graph.

### Transwell migration and invasion assay

Cell migration and invasion capabilities were evaluated using Transwell assays with 8 μm-pore Boyden chambers (Corning Incorporated, NY, USA) in 24-well plates. Briefly, cells were plated in the upper chamber, either uncoated (for migration assay) or precoated with Matrigel (for invasion assay; Corning, NY, USA). After 48 hours of incubation, non-migratory or non-invaded cells were removed from the upper surface with a cotton swab. Cells that had traversed the membrane were fixed with 4 % formaldehyde for 10 minutes, stained with 0.5 % crystal violet, and imaged under an inverted microscope (Olympus). The number of migrated or invaded cells was quantified from five randomly selected fields per sample at 200 × magnification. All experiments were performed in triplicate.

### Transwell coculture assay

Coculture experiments were conducted using 6-well Boyden chamber inserts with 0.4 μm pores (Corning Incorporated, NY, USA). Prior to coculture, THP-1 cells were induced with 320 nM PMA for 24 hours to differentiate into M0 macrophages (THP-1-M0). A total of 1 × 10⁶ THP-1-M0 or iBMDM cells were plated in the lower chamber, while 5 × 10⁵ MDA-MB-231 or 4T1 cells were seeded in the upper chamber of the Transwell system and cocultured for 48 hours. Following incubation, macrophages and corresponding culture supernatants were separately collected for subsequent analysis via quantitative real-time PCR (qRT-PCR), flow cytometry (FCM), and enzyme-linked immunosorbent assay (ELISA).

### Western blot and co-immunoprecipitation (Co-IP) assay

Total cellular proteins were extracted using lysis buffer supplemented with protease and phosphatase inhibitors (Beyotime, Shanghai, China) for Western blotting and immunoprecipitation. Protein concentration was determined using a bicinchoninic acid (BCA) assay. Lysates were separated by sodium dodecyl sulfate–polyacrylamide gel electrophoresis (SDS-PAGE) and transferred to PVDF membranes, which were then incubated with specified primary and secondary antibodies. Protein bands were visualized by enhanced chemiluminescence using ECL prime substrate. For co-immunoprecipitation (Co-IP), 10 % of the total protein lysate was reserved as input. The remaining lysates were incubated overnight at 4 °C with primary antibodies coupled to protein A/G beads. The immunoprecipitated complexes were washed three times and subsequently analyzed by Western blotting. Details regarding all antibodies used are provided in Table S2 (Supporting Information).

### LC-MS/MS analysis

Liquid chromatography-tandem mass spectrometry (LC-MS/MS) was employed to identify potential interacting proteins of the target protein. Briefly, precipitated proteins were resolved on 7.5 % gradient gels and visualized using silver staining (Beyotime, Shanghai, China). Target protein bands were excised, subjected to in-gel digestion, and peptides were extracted. The resulting peptides were mixed with matrix solution and spotted onto a MALDI target plate. LC-MS/MS analysis of SNO-modified peptides was performed on a NanoLC system coupled to a TripleTOF® 5600+ mass spectrometer (AB Sciex). Raw MS/MS spectra were processed and searched against the UniProt human proteome database using SequestHT, with a variable modification of 451.54 Da (corresponding to Biotin-maleimide) applied to cysteine residues. Database search results were filtered to achieve a false discovery rate (FDR) of <1 % at both the peptide and protein levels.

### Immunohistochemistry (IHC) assay

Immunohistochemical staining was performed on triple-negative breast cancer (TNBC) tissue specimens. Briefly, formalin-fixed, paraffin-embedded tissues were sectioned at a thickness of 4 μm. Sections were deparaffinized, rehydrated, and subjected to antigen retrieval, followed by quenching of endogenous peroxidase activity. The samples were then incubated with primary antibodies overnight at 4 °C. After washing, the sections were treated with an HRP-conjugated secondary antibody. Immunodetection was carried out using a 3,3′-diaminobenzidine (DAB) substrate, and counterstaining was performed with hematoxylin. Staining results were evaluated independently by two blinded pathologists without knowledge of clinical outcomes.

### Immunofluorescence staining

For immunofluorescence analysis, cells were plated onto confocal dishes. Following incubation, cells were rinsed with phosphate-buffered saline (PBS), fixed with 4 % paraformaldehyde for 15 minutes, and permeabilized with 0.1 % Triton X-100 for 10 minutes at room temperature. Non-specific binding sites were blocked with 0.5 % bovine serum albumin (BSA) for 1 h. Samples were then incubated with primary antibodies overnight at 4 °C, followed by incubation with appropriate fluorescently labeled secondary antibodies. Subcellular localization was examined using an Olympus FV3000RS confocal laser scanning microscope (Olympus, Tokyo, Japan).

### Proximity ligation assay (PLA)

The Proximity Ligation Assay was performed according to the manufacturer’s protocol using the NaveniFlex Cell MR kit (Sigma-Aldrich, Sweden). Briefly, cells grown on confocal dishes were fixed with 4 % paraformaldehyde in PBS for 15 minutes at room temperature. After fixation, samples were washed with TBST and then blocked with Blocking Solution for 60 minutes at 37 °C in a preheated humidified chamber. Following blocking, the solution was removed and replaced with primary antibodies diluted in Antibody Diluent, followed by incubation for 60 minutes at 37 °C. After washing with TBST, the proximity ligation reaction was carried out using the NaveniFlex Cell MR kit. Nuclei were counterstained with DAPI. PLA signals were visualized using an Olympus FV3000RS confocal laser scanning microscope (Olympus, Tokyo, Japan) under a 40 × objective.

### Ubiquitination and protein half-life assay

To assess deubiquitination *in vivo*, OTUD4-knockdown cells were treated with the proteasome inhibitor MG132 (10 µM) for 6–8 hours prior to harvesting and subsequently subjected to analysis of ZMYND8 ubiquitination levels. For *in vitro* deubiquitination assays, HEK-293T cells were co-transfected with plasmids encoding His-ZMYND8, Flag-OTUD4, and HA-ubiquitin (Ub), followed by MG132 (10 µM) treatment for 6–8 hours before collection to evaluate ZMYND8 ubiquitination. To determine protein half-life, cells were treated with the protein synthesis inhibitor cycloheximide (CHX) and harvested at indicated time points in a temporal gradient. Protein levels were analyzed by Western blotting and quantified using ImageJ software.

### Animal studies

Six- to eight-week-old female BALB/c mice were obtained from Charles River Laboratories (Zhejiang, China). For the orthotopic mammary fat pad injection model, mice were inoculated in the fourth mammary fat pad with 2.5 × 10⁶ luciferase-expressing ZMYND8-KO or ZMYND8-OE 4T1 cells suspended in 100 μL of PBS. Once tumor volume reached approximately 1,000 mm³, the mice were euthanized and primary tumors were harvested. Tumor volume was calculated according to the formula: tumor volume (mm^3^) = 0.5 × length × width^2^. For the intracardiac injection spinal metastasis model, mice were injected intracardially with 1 × 10⁵ luciferase-expressing ZMYND8-OE 4T1 cells in 100 μL of PBS. Three days post-injection, mice were administered the luciferase substrate d-luciferin intraperitoneally and imaged using an IVIS Lumina III Ultra Resolution *in vivo* imaging system.

### Statistical analysis

Statistical analyses were conducted using GraphPad Prism software (version 9.0). Comparisons between two groups were performed using the two-tailed Student’s t-test. For comparisons among multiple groups, one- or two-way ANOVA was applied followed by appropriate multiple comparisons tests. Survival analysis was carried out using Kaplan–Meier curves, and differences were assessed with the log-rank test. The number of biological replicates (n) for each experiment is indicated in the figures or corresponding legends. Each biological replicate *in vitro* included two to three technical replicates. Data are presented as mean ± SEM. Differences were considered statistically significant at *p* < 0.05.

## Results

### ZMYND8 protein is upregulated in human spinal metastasis of triple-negative breast cancer (TNBC)

**Fig. 1:**
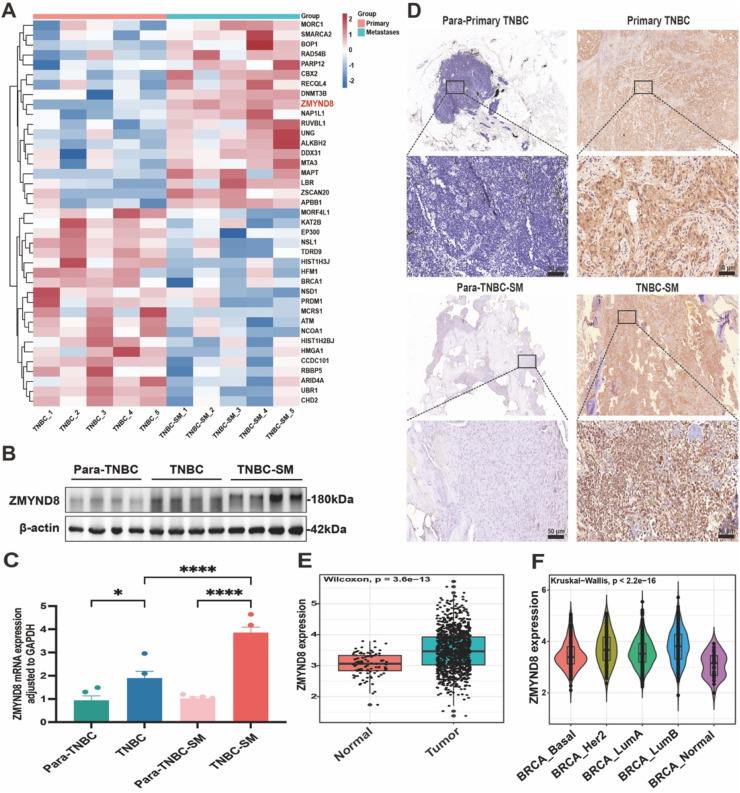
Elevated ZMYND8 Expression in Primary and Spinal Metastatic TNBC Tissues Correlates with Disease Progression. (A) Heatmap displaying differentially expressed genes between primary triple-negative breast cancer (TNBC) and TNBC spinal metastasis (TNBC-SM) tissues. (B) Western blot analysis of ZMYND8 protein expression in primary TNBC, TNBC-SM, and corresponding adjacent normal tissues. (C) Relative mRNA expression levels of ZMYND8 in the aforementioned tissues, as determined by RT-qPCR. (D) Representative immunohistochemical (IHC) staining images showing ZMYND8 expression across different tissue types; scale bar: 50 μm. (E, F) Comparative analysis of ZMYND8 expression in normal breast tissue and various molecular subtypes of breast cancer (BRCA_Basal, BRCA_Her2, BRCA_LumA and BRCA_LumB). *p < 0.05, **p < 0.01, ***p < 0.001, ****p < 0.0001.

Although previous studies have reported the overexpression of ZMYND8 in breast cancer (BRCA), its expression in advanced breast cancer, particularly in TNBC-SM, remains unexplored. In this study, high-throughput RNA sequencing was performed to identify differentially expressed genes in primary breast cancer tissues and spinal metastatic lesions from five patients with TNBC and five with TNBC-SM. As shown in Fig. 1A, ZMYND8 expression was significantly higher in the TNBC-SM group. Further analysis revealed that ZMYND8 expression was markedly elevated at both the mRNA and protein levels in human spinal metastatic tissues compared to primary breast cancer lesions and matched adjacent non-tumor tissues (Fig. 1B, C). This finding was further confirmed by immunohistochemical (IHC) staining (Fig. 1D; Fig. S1, Supporting Information). IHC results also demonstrated that ZMYND8 expression was higher in spinal metastatic lesions than in primary breast cancer tissues. Moreover, ZMYND8 was rarely expressed in adjacent normal tissues. The low expression of ZMYND8 in normal tissues and its high expression in the tumor microenvironment (TME) suggest the potential for tumor-targeted blockade therapy against ZMYND8.

Data mining of the publicly available BRCA cohort from the TCGA database revealed that ZMYND8 expression was significantly higher in breast cancer tissues than in normal tissues, indicating its potential role in breast cancer pathogenesis (Fig. 1E). Differences in ZMYND8 expression were observed across various breast cancer subtypes, suggesting its association with subtype-specific biological characteristics (Fig. 1F). Further analysis of the relationship between ZMYND8 expression and clinicopathological features showed that high ZMYND8 expression was positively correlated with advanced tumor stage and lymph node metastasis, implying its involvement in breast cancer progression (Fig. S2A, B, Supporting Information).

To evaluate whether ZMYND8 is associated with breast cancer bone metastasis, bone metastasis-related genes were screened from the MSigDB database, and correlation analyses were conducted. The results indicated that ZMYND8 expression was significantly positively correlated with multiple bone metastasis-related genes, and these genes were markedly upregulated in the ZMYND8 high-expression group (Fig. S3A, B, Supporting Information). These findings suggest that ZMYND8 may facilitate breast cancer bone metastasis by regulating the expression of bone metastasis-related genes, highlighting its potential as a therapeutic target for inhibiting bone metastasis in breast cancer.

### Elevated ZMYND8 expression is correlated with an immune-inert phenotype and poor prognosis in human spinal metastasis of triple-negative breast cancer (TNBC)

The tumor microenvironment (TME) plays a critical role in shaping cancer metastasis and determining therapeutic response. Through bioinformatic analysis of transcriptomic data from patients with TNBC and TNBC-SM, we found that the TME of metastatic breast cancer exhibits a more immunologically inert phenotype compared to that of primary tumors. However, how tumor cells contribute to the establishment of such a TME remains unclear.

**Fig. 2:**
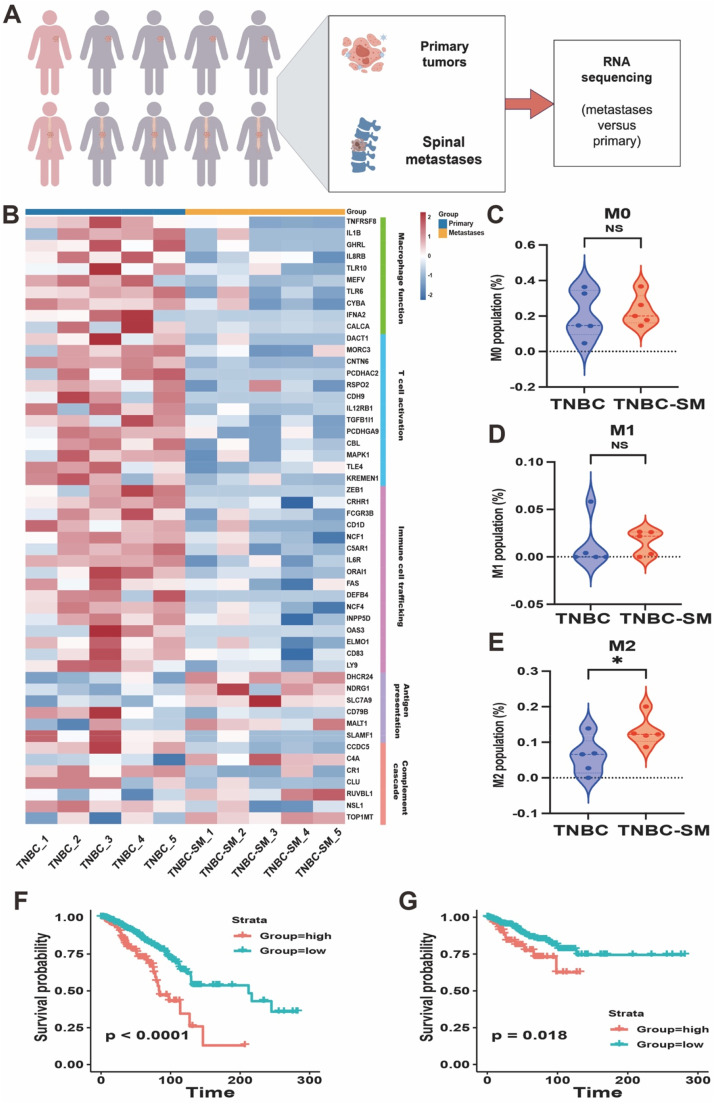
Elevated ZMYND8 Expression Is Associated with an Immunosuppressive Microenvironment and Poor Prognosis in Triple-Negative Breast Cancer Spinal Metastasis (TNBC-SM). (A) Schematic diagram of the study design illustrating the collection of primary breast cancer and matched spinal metastatic tissues from patients for RNA sequencing and comparative analysis of immune-related gene expression profiles. (B) Heatmap demonstrating the expression patterns of immune-related genes in primary TNBC and TNBC-SM tissues. (C–E) CIBERSORTx analysis of RNA-Seq data from five paired primary and spinal metastatic breast cancer samples was performed to characterize the immune cell composition. The ratios of M1 and M2 macrophages to total macrophages are shown. A significant increase in M2 macrophages was observed in metastatic lesions, suggesting their potential role in facilitating immune evasion and tumor progression. (F, G) Kaplan–Meier analysis of overall survival (OS) and disease-free survival (DFS) based on ZMYND8 expression in TCGA TNBC patients. *p < 0.05, **p < 0.01, ***p < 0.001, ****p < 0.0001.

To better characterize differences in the immune microenvironment and identify regulators of the tumor immune landscape and drivers of metastasis, we compared transcriptomic data from five matched pairs of primary and spinal metastatic triple-negative breast tumor samples (Fig. 2A). Compared to matched primary tumors, most immune-related genes were downregulated in metastatic lesions, particularly those involved in macrophage function and T-cell activation (Fig. 2B). To further investigate differences in immune cell composition between matched primary and metastatic microenvironments, we analyzed RNA-Seq data using CIBERSORTx. Interestingly, the proportion of M2 macrophages was significantly increased in the TNBC-SM group (*p* < 0.05; Fig. 2C-E). In contrast, the proportions of M1 macrophages and total macrophages did not differ significantly between primary and matched metastatic tumors. These results suggest that shifts in macrophage polarization, specifically an increase in M2 macrophages, represent a major immunological distinction between the TME of primary and metastatic breast tumors in our dataset.

Finally, we assessed cumulative survival outcomes based on ZMYND8 expression. As shown in Fig. 2F, G, patients with high ZMYND8 expression exhibited significantly reduced overall survival (OS) and disease-free survival (DFS). These findings indicate a significant correlation between elevated ZMYND8 expression and an immunosuppressive TME as well as unfavorable prognosis in TNBC.

### ZMYND8 promotes triple-negative breast cancer (TNBC) cell invasion, migration, and metastasis

**Fig. 3:**
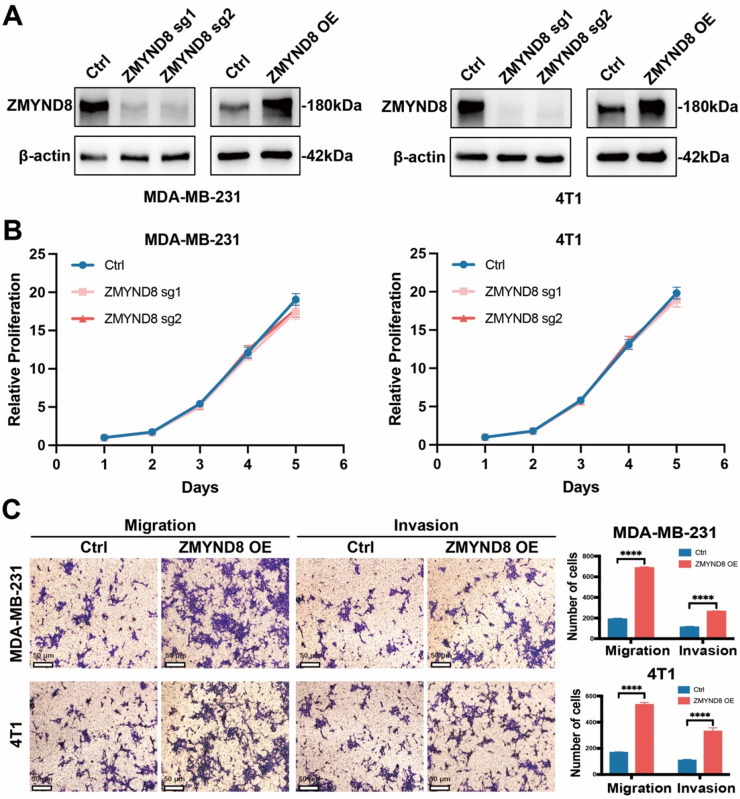
ZMYND8 Promotes Invasion, Migration, and Metastasis in Triple-Negative Breast Cancer (TNBC) Cells. (A) Western blot analysis of ZMYND8 protein levels in MDA-MB-231 and 4T1 cell lines. The β-actin protein was used as a reference control. (B) CCK-8 assay evaluating the effect of ZMYND8 on proliferative capacity in MDA-MB-231 and 4T1 breast cancer cells. Data in (Β) are presented as mean ± SD and analyzed by two-way analysis of variance (ANOVA) test. (C) Transwell migration and invasion assays assessing the influence of ZMYND8 on invasive and migratory capabilities in MDA-MB-231 and 4T1 cells; scale bar: 50 μm. Data in (C) are presented as mean ± SD and analyzed by Student’s t-test. *, *p* < 0.05; **, *p* < 0.01; ***, *p* < 0.001; ****, *p* < 0.0001. Data shown are representative of three independent experiments.

To investigate the role of ZMYND8 in the progression of triple-negative breast cancer, we examined its effects on cell proliferation, migration, and invasion *in vitro*. Knockout and overexpression models of ZMYND8 were generated in MDA-MB-231 and 4T1 cells using the CRISPR/Cas9 and lentiviral systems, with transfection efficiency confirmed by western blotting (Fig. 3A). CCK-8 assays revealed no statistically significant difference in proliferative activity between ZMYND8-knockout breast cancer cells (ZMYND8-sg1, ZMYND8-sg2) and the control group (Ctrl) (Fig. 3B). Transwell assay results demonstrated that under both non-Matrigel (migration) and Matrigel-coated (invasion) conditions, ZMYND8-overexpressing breast cancer cells exhibited significantly enhanced migratory and invasive capabilities (Fig. 3C). These results indicate that ZMYND8 knockout does not affect breast cancer cell proliferation *in vitro*, whereas its overexpression promotes cell migration and invasion.

### ZMYND8 promotes malignant progression and an immunosuppressive microenvironment in triple-negative breast cancer

**Fig. 4:**
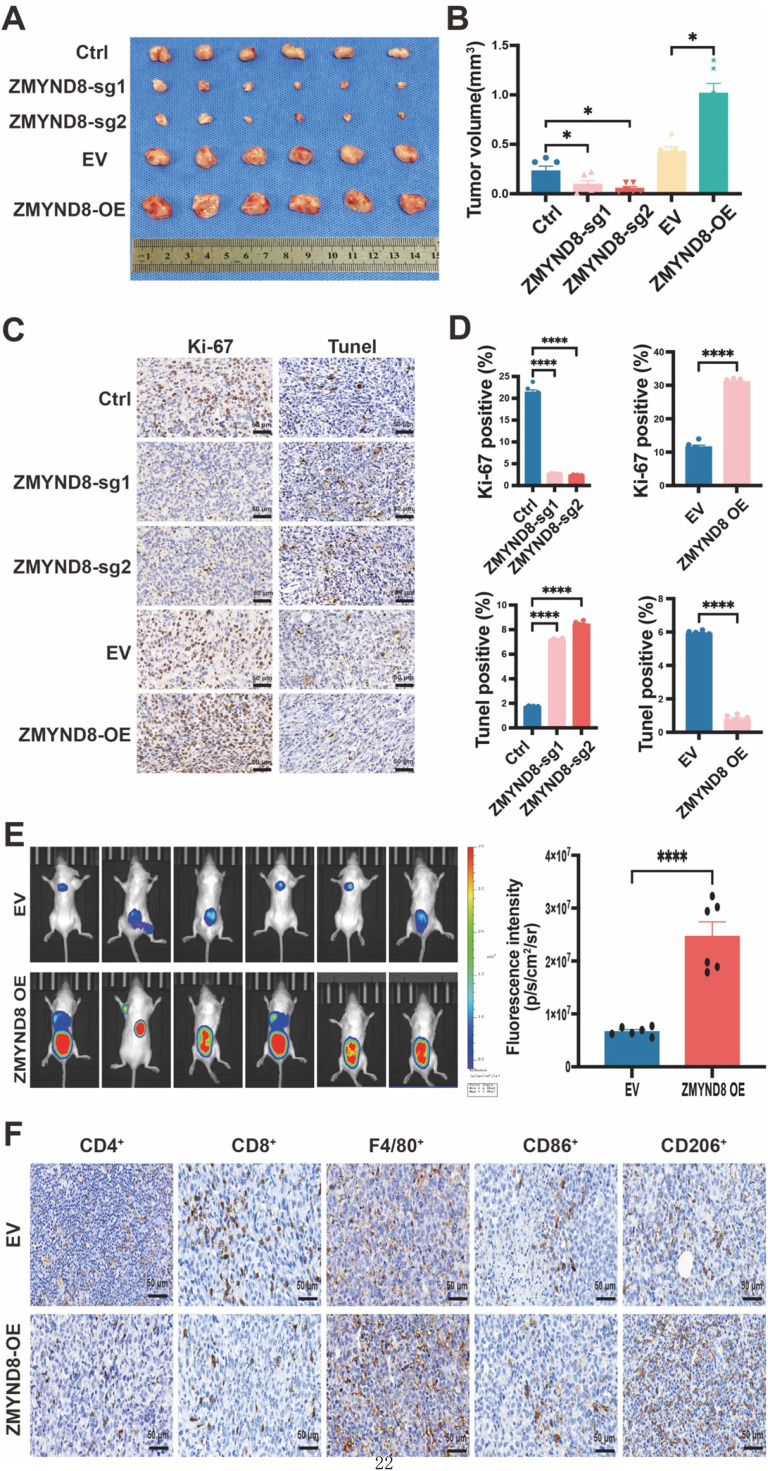
ZMYND8 Promotes Malignant Progression and an Immunosuppressive Microenvironment in Triple-Negative Breast Cancer In Vivo. (A, B) Orthotopic xenograft model evaluating the effect of differential ZMYND8 expression on tumor growth using 4T1 cells. Data in (Β) are presented as mean ± SD and analyzed by one-way ANOVA. (C, D) Representative images of Ki-67 and TUNEL staining in tumor tissues, analyzing the proliferation and apoptosis status of orthotopic tumors *in vivo*; scale bar: 50 μm. Data in (D) are presented as mean ± SD and analyzed by one-way ANOVA. (E) Bioluminescence imaging and statistical analysis of spinal metastasis in BALB/c mice following intracardiac injection of control and ZMYND8-overexpressing 4T1 cells. Data in (E) are presented as mean ± SD and analyzed by Student’s t-test. (F) Immunohistochemical detection of CD4⁺, CD8⁺, F4/80⁺, CD86⁺, and CD206⁺ cells in 4T1-derived mouse breast cancer tissues; scale bar: 50 μm. *, *p* < 0.05; **, *p* < 0.01; ***, *p* < 0.001; ****, *p* < 0.0001.

To investigate the effect of ZMYND8 on breast cancer growth *in vivo*, stable ZMYND8-knockout and overexpressing 4T1 cell lines were established. Using an orthotopic breast cancer model, we found that low ZMYND8 expression significantly reduced primary tumor growth in BALB/c mice, suggesting that ZMYND8 promotes breast tumor growth (Fig. 4A, B). Notably, this effect was not attributable to changes in the intrinsic proliferative capacity of tumor cells, as ZMYND8 knockout did not alter breast cancer cell proliferation *in vitro* (Fig. 3B). Because *in vivo* tumor growth is shaped not only by the intrinsic proliferative capacity of cancer cells but also by a variety of microenvironmental factors, including apoptosis, immune infiltration, stromal interactions, and cytokine-mediated signaling, we further assessed Ki-67 and TUNEL staining in orthotopic tumor tissues. Tumors from ZMYND8-overexpressing cells exhibited increased Ki-67 positivity and reduced TUNEL positivity compared with controls, reflecting enhanced proliferative activity and reduced apoptosis within the *in vivo* tumor microenvironment (Fig. 4C, D). These findings characterize how ZMYND8-driven differences in tumor growth manifest *in vivo* despite unchanged proliferation *in vitro*. Together, these results support a pro-tumorigenic role for ZMYND8 in TNBC progression.

To evaluate whether ZMYND8 mediates spinal metastasis in breast cancer, a mouse spinal metastasis model was established. Tumor progression was monitored using *in vivo* bioluminescence imaging (BLI). Representative BLI images showed typical spinal metastatic lesions in both groups of mice on day 21 (Fig. 4E). Statistical analysis revealed that ZMYND8 upregulation increased both the incidence of spinal metastasis and tumor burden in mice. Thus, these results demonstrate that ZMYND8 promotes spinal metastasis of TNBC *in vivo*.

Given the association between high ZMYND8 expression and an immunosuppressive microenvironment in breast cancer (Fig. 2), we further investigated the function and regulatory mechanisms of ZMYND8 in the TNBC TME using a tumor-bearing model. Immunohistochemical results showed a significant increase in the infiltration of F4/80⁺ and CD206⁺ macrophages, along with a notable reduction in CD4⁺ and CD8⁺ T cell infiltration, in ZMYND8-overexpressing tumors (Fig. 4F; Fig. S4, Supporting Information). These findings suggest that high ZMYND8 expression remodels the TME toward an immunosuppressive state by enhancing TAM infiltration and inhibiting T cell recruitment.

### ZMYND8 induces recruitment and M2-like polarization of macrophages

**Fig. 5:**
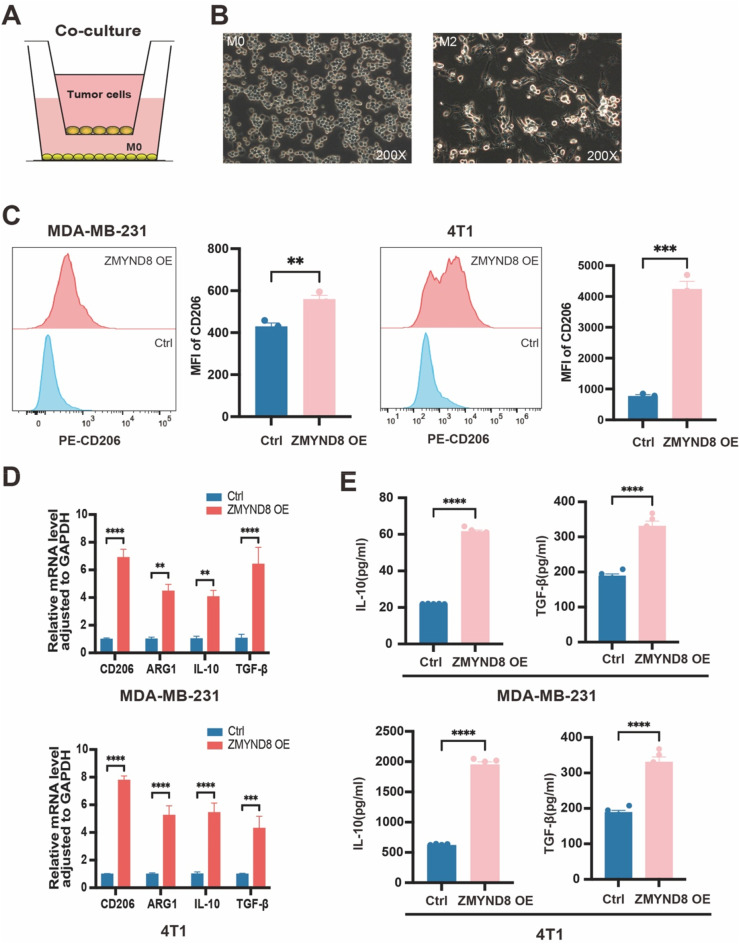
ZMYND8 Induces Recruitment and M2-like Polarization of Macrophages. (A) Schematic diagram of the indirect coculture system between breast cancer cells and macrophages. (B) Altered macrophage morphology with pseudopodia formation and spindle-like features was observed under a microscope after coculture with breast cancer cells. (C) Flow cytometric analysis of the M2 marker CD206 on macrophages following coculture. (D) RT-qPCR analysis of M2-associated genes (CD206, ARG1, IL10, TGF-β) in macrophages. (E) ELISA measurement of IL-10 and TGF-β1 secretion levels in the coculture system. Data in (C-E) are presented as mean ± SD and analyzed by Student’s t-test. *, *p* < 0.05; **, *p* < 0.01; ***, *p* < 0.001; ****, *p* < 0.0001.

Elevated expression of ZMYND8 has been shown to induce an immunosuppressive microenvironment in breast cancer, wherein M2-like tumor-associated macrophages (TAMs) play a critical role. Based on this, we hypothesized that ZMYND8 in breast cancer cells may promote immunosuppression by regulating macrophage recruitment and polarization. To test this hypothesis, an *in vitro* co-culture system was employed to evaluate the effect of ZMYND8 expression on macrophages. THP-1 cells were treated with phorbol-12-myristate-13-acetate (PMA) to differentiate them into M0 macrophages. After co-culture with ZMYND8-overexpressing (OE) TNBC cells (Fig. 5A, B), macrophages exhibited significantly upregulated expression of the M2 surface marker CD206, increased mRNA levels of CD206, ARG-1, IL-10, and TGF-β, and produced higher levels of IL-10 and TGF-β1 (Fig. 5C-E). Transwell assays further demonstrated that ZMYND8-OE breast cancer cells promoted the chemotactic migration of macrophages (Fig. S5, Supporting Information). Together, these results indicate that ZMYND8-overexpressing TNBC cells facilitate macrophage recruitment and M2-like polarization.

### ZMYND8 acts as a scaffold to strengthen the DDX3X-CK1ε interaction and activates WNT/β-catenin signaling

**Fig. 6:**
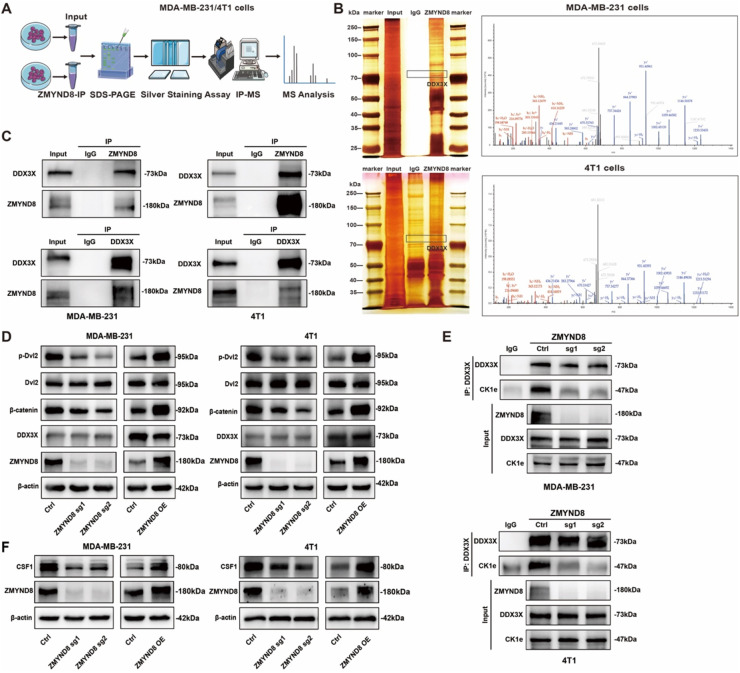
ZMYND8 Directly Binds DDX3X and CK1ε to Enhance Their Interaction and Activate the WNT/β-Catenin Signaling Pathway. (A) Schematic overview of the quantitative proteomic screening strategy used to identify ZMYND8-interacting proteins. (B) Endogenous ZMYND8 was immunoprecipitated from MDA-MB-231 and 4T1 cell lysates. Co-precipitated proteins were separated by SDS-PAGE and visualized by silver staining, followed by identification via mass spectrometry. DDX3X was identified as a ZMYND8 binding partner; representative spectra are shown. (C) Endogenous interaction between ZMYND8 and DDX3X was examined in MDA-MB-231 and 4T1 cells via co-immunoprecipitation and immunoblotting with indicated antibodies. (D) Western blot analysis of the effect of ZMYND8 on key components of the WNT/β-catenin signaling pathway. (E) Co-immunoprecipitation analysis of the DDX3X–CK1ε complex in control and ZMYND8-knockout MDA-MB-231 and 4T1 cells. Input panels show the validated loss of ZMYND8 protein in both knockout cell lines. The interaction between DDX3X and CK1ε was reduced upon ZMYND8 depletion. (F) Regulation of CSF1 expression by ZMYND8 as determined by Western blot. Data shown are representative of three independent experiments.

To further investigate the molecular mechanism of ZMYND8 in triple-negative breast cancer spinal metastasis, we performed KEGG pathway analysis on the ZMYND8-regulated transcriptome, which suggested a potential role of ZMYND8 in modulating the WNT signaling pathway (Fig. S6, Supporting Information). Based on this, subsequent studies focused on the mechanism of the WNT pathway in ZMYND8-mediated macrophage recruitment and polarization. To explore the molecular mechanism by which ZMYND8 regulates WNT/β-catenin signaling, immunoprecipitation combined with silver staining and mass spectrometry (IP–MS) was employed to screen and identify DDX3X as a potential co-chaperone of ZMYND8 (Fig. 6A, B; Fig. S7, Supporting Information). Notably, DDX3X is known to interact with CK1ε and to stimulate its kinase activity, thereby promoting the phosphorylation of DVL (Dishevelled family proteins), driving β-catenin nuclear import and activating WNT/β-catenin signaling [[44], [45], [46]]. This finding provides new clues for understanding the role of ZMYND8 in WNT/β-catenin signal transduction. Given that the binding between DDX3X and CK1ε is known to induce DVL phosphorylation, enhancing nuclear accumulation of β-catenin and activation of WNT/β-catenin signaling [45], we next sought to determine whether the interaction of ZMYND8 with DDX3X and CK1ε is required for activation of this pathway. As shown in Fig. 6C, endogenous co-immunoprecipitation (Co-IP) analysis confirmed an interaction between ZMYND8 and DDX3X. Furthermore, to investigate whether ZMYND8 expression influences DDX3X protein levels via this interaction, western blot analysis showed that altering ZMYND8 expression did not affect DDX3X protein levels (Fig. 6D). To clarify how ZMYND8 affects WNT/β-catenin signaling through its interaction with DDX3X, Co-IP experiments were conducted to examine whether ZMYND8 enhances the interaction between DDX3X and CK1ε, thereby promoting pathway activation. As shown in Fig. 6E, Co-IP results demonstrated that the level of the DDX3X–CK1ε complex was reduced in the ZMYND8-knockout group compared to the control. Together, these results indicate that ZMYND8 directly binds DDX3X and strengthens the DDX3X–CK1ε interaction, facilitating DVL phosphorylation and activation of WNT/β-catenin signaling.

Given the critical role of CSF1 in macrophage recruitment and M2 polarization [47,48], it was selected as a validation target. Western blot results showed that ZMYND8 overexpression upregulated CSF1 protein expression, while its knockout downregulated CSF1 (Fig. 6F). These results suggest that ZMYND8 promotes macrophage recruitment and M2-like polarization by enhancing CSF1 expression in triple-negative breast cancer cells.

### OTUD4 interacts with and stabilizes ZMYND8

**Fig. 7:**
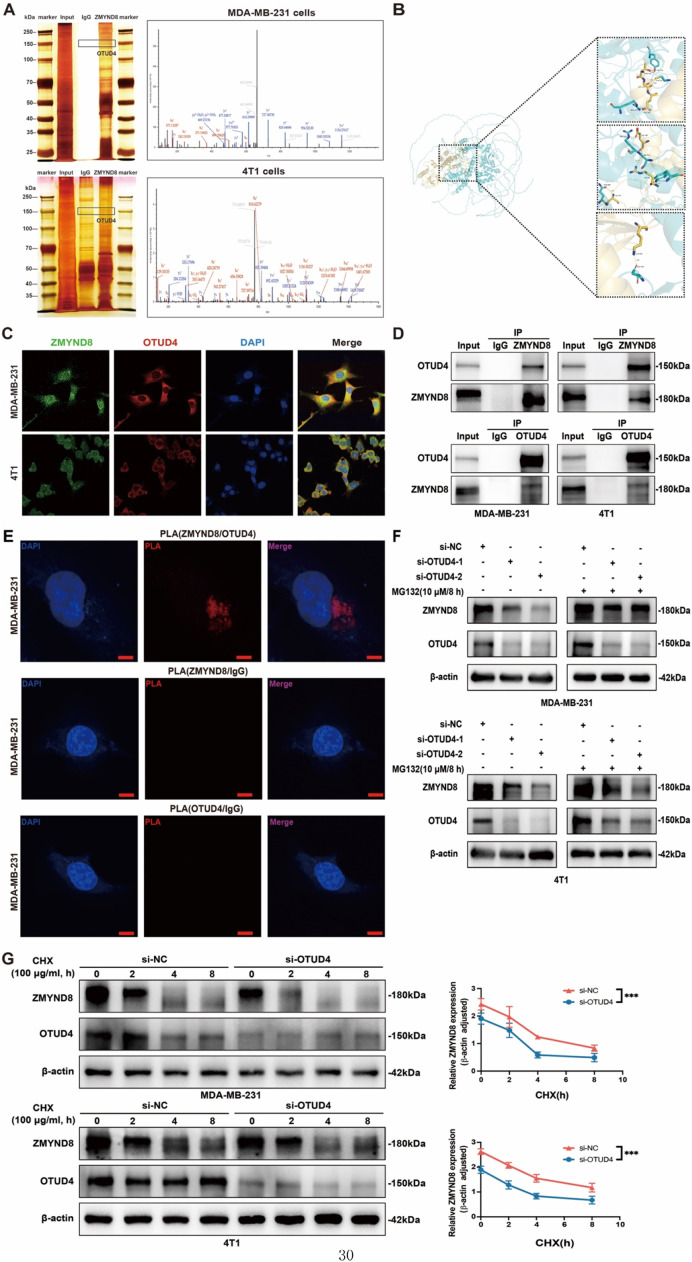
OTUD4 Directly Interacts with and Stabilizes ZMYND8. (A) Endogenous ZMYND8 was immunoprecipitated from MDA-MB-231 and 4T1 cell lysates. Associated proteins were separated by SDS-PAGE, visualized by silver staining, and identified by mass spectrometry. OTUD4 was identified as a ZMYND8-binding partner; representative spectral data are shown. (B) Predicted interaction between OTUD4 and ZMYND8 using RosettaDock modeling. (C) Confocal microscopy images showing colocalization of OTUD4 and ZMYND8 in MDA-MB-231 and 4T1 cells. Scale bar: 10 μm. (D) Endogenous interaction between OTUD4 and ZMYND8 was examined in MDA-MB-231 and 4T1 cells by co-immunoprecipitation and immunoblotting with indicated antibodies. (E) Proximity ligation assay (PLA) detecting the interaction between ZMYND8 and OTUD4 in MDA-MB-231 cells. Red fluorescent dots indicate positive protein–protein interactions; IgG was used as a negative control. Scale bar: 10 μm. (F) Western blot analysis of ZMYND8 protein levels in MDA-MB-231 and 4T1 cells following OTUD4 knockdown and treatment with MG132 (10 μM, 8 h). (G) ZMYND8 protein stability was assessed by Western blot in OTUD4-knockdown MDA-MB-231 and 4T1 cells treated with cycloheximide (CHX, 100 μg/mL) for indicated durations. Data in (H) are presented as mean ± SD and analyzed by two-way ANOVA. *, *p* < 0.05; **, *p* < 0.01; ***, *p* < 0.001; ****, *p* < 0.0001. Data shown are representative of three independent experiments.

To identify potential regulators of ZMYND8 protein abundance, immunoprecipitation combined with silver staining and mass spectrometry (IP–MS) was performed in human MDA-MB-231 and murine 4T1 TNBC cells. This unbiased screen revealed several deubiquitinases in ZMYND8-containing complexes; among them, OTUD4 ranked as one of the top ZMYND8-associated DUBs based on peptide counts and spectral abundance, whereas USP7 was not detected in MDA-MB-231 cells and appeared at much lower abundance than OTUD4 in 4T1 cells. These data prompted us to focus subsequent validation on OTUD4. The results indicated an interaction between ZMYND8 and the deubiquitinase OTUD4 (Fig. 7A). Given that OTUD4 functions as a deubiquitinating enzyme that removes ubiquitin modifications from its target proteins, we hypothesized that OTUD4 might induce the observed phenotypes by interacting with and deubiquitinating ZMYND8. RosettaDock analysis predicted a direct interaction between ZMYND8 and OTUD4 (Fig. 7B). Although both OTUD4 and ZMYND8 exhibit cytoplasmic and nuclear distribution, confocal microscopy revealed predominant cytoplasmic colocalization of ZMYND8 and OTUD4 in MDA-MB-231 and 4T1 cells (Fig. 7C). Consistent with this hypothesis, endogenous co-immunoprecipitation (Co-IP) assays in MDA-MB-231 and 4T1 cells confirmed the interaction between ZMYND8 and OTUD4 (Fig. 7D). Furthermore, *in situ* proximity ligation assay (PLA) validated the direct *in situ* interaction between ZMYND8 and OTUD4 (Fig. 7E).

As a deubiquitinase, OTUD4 regulates protein stability by removing ubiquitin modifications from substrate proteins through its deubiquitinating activity [49]. Co-IP experiments demonstrated that endogenous ZMYND8 interacts with OTUD4, suggesting that OTUD4 may regulate ZMYND8 protein stability. To test this hypothesis, OTUD4 was knocked down using siRNA in MDA-MB-231 and 4T1 breast cancer cells. Western blot analysis showed that ZMYND8 protein levels were significantly reduced upon OTUD4 knockdown (Fig. 7F). Subsequent treatment with the proteasome inhibitor MG132 (10 μM, 8 h) partially restored ZMYND8 protein expression, indicating that OTUD4 maintains ZMYND8 stability by inhibiting proteasome-mediated degradation (Fig. 7F). To further evaluate the specific regulatory role of OTUD4 in ZMYND8 stability, control and OTUD4-knockdown cells (MDA-MB-231 and 4T1) were treated with the protein synthesis inhibitor cycloheximide (CHX). ZMYND8 protein levels were analyzed by western blot at 0, 2, 4, and 8 hours, and a degradation curve was plotted to estimate its half-life. The results showed that OTUD4 knockdown significantly accelerated ZMYND8 degradation, with a pronounced decline after 4 hours (Fig. 7G), indicating that OTUD4 extends the half-life and enhances the stability of ZMYND8. In summary, these findings demonstrate that OTUD4 maintains stable expression of ZMYND8 protein through its deubiquitinating activity.

### OTUD4 deubiquitinates ZMYND8 in vivo and in vitro

**Fig. 8:**
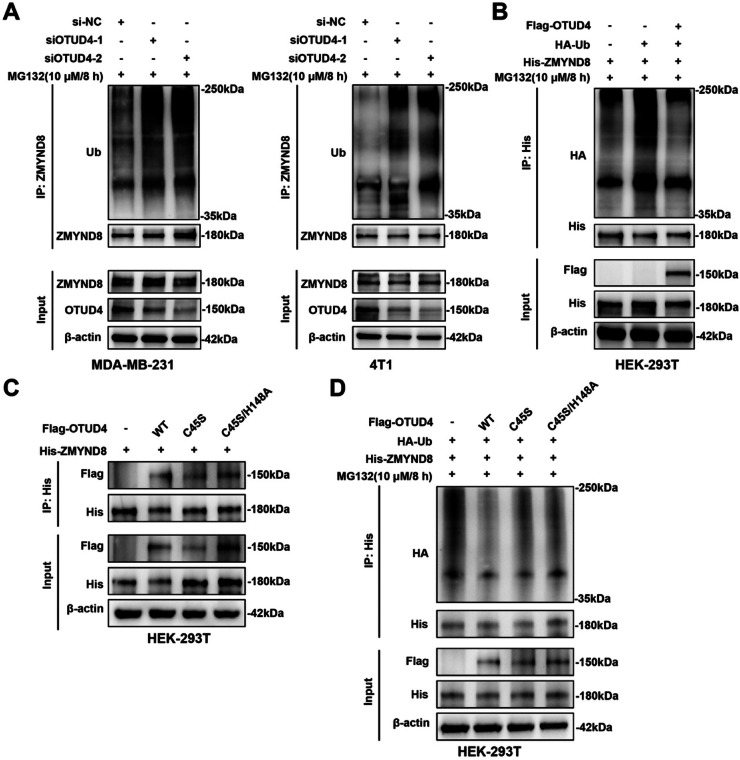
OTUD4 Deubiquitinates ZMYND8 in Vivo and in Vitro. (A) Ubiquitination levels of ZMYND8 in MDA-MB-231 and 4T1 cells following OTUD4 knockdown were examined by immunoprecipitation with an anti-ZMYND8 antibody and immunoblotting with an anti-ubiquitin antibody. (B) HEK-293T cells were co-transfected with plasmids encoding HA-Ub, Flag-OTUD4, and His-ZMYND8 for 72 hours and treated with 10 μM MG132 for 8 hours before harvesting. Cell lysates were immunoprecipitated with an anti-His antibody, and ZMYND8 ubiquitination was detected by immunoblotting using an anti-HA antibody. (C) Interaction analysis between ZMYND8 and OTUD4 catalytic-inactive mutants. HEK-293T cells were co-transfected with plasmids encoding His-ZMYND8 and either wild-type OTUD4 or catalytic-inactive OTUD4 variants (C45S or C45S/H148A). Cell lysates were immunoprecipitated with an anti-His antibody, and the presence of OTUD4 in the immunocomplexes was assessed using an anti-Flag antibody. Both catalytic-inactive mutants retained the ability to interact with ZMYND8. (D) Ubiquitination assay evaluating the catalytic requirement of OTUD4 in ZMYND8 deubiquitination. HEK-293T cells were co-transfected with plasmids encoding His-ZMYND8, HA-Ub, and either wild-type OTUD4 or catalytic-inactive OTUD4 mutants (C45S or C45S/H148A), followed by treatment with 10 μM MG132 for 8 hours. Cell lysates were immunoprecipitated with an anti-His antibody, and ZMYND8 ubiquitination was detected using an anti-HA antibody. Catalytic-inactive OTUD4 variants failed to reduce ZMYND8 ubiquitination, confirming that OTUD4 enzymatic activity is required for ZMYND8 deubiquitination. Data shown are representative of three independent experiments.

As OTUD4 is a well-established deubiquitinase (DUB) and regulates the stability of ZMYND8 (Fig. 7), we conducted both *in vivo* and *in vitro* ubiquitination assays to determine whether OTUD4 controls ZMYND8 turnover via ubiquitination. As shown in Fig. 8A, knockdown of OTUD4 significantly enhanced ubiquitination of ZMYND8 in MDA-MB-231 and 4T1 cells, suggesting that OTUD4 negatively regulates the ubiquitination level of ZMYND8. Furthermore, co-transfection of His-ZMYND8, Flag-OTUD4, and HA-Ub plasmids in HEK-293T cells demonstrated that overexpression of OTUD4 markedly reduced ZMYND8 ubiquitination (Fig. 8B), confirming that OTUD4 promotes ZMYND8 stability through a deubiquitination mechanism.

To further determine whether the deubiquitinating activity of OTUD4 is required for the removal of ubiquitin from ZMYND8, we generated catalytic-inactive OTUD4 mutants based on previously reported structural and biochemical analyses of OTU family deubiquitinases[42,50,51]. Cys45 and His148 are known to constitute the core catalytic residues of OTUD4, forming the nucleophilic and general-base components of the conserved catalytic triad. Mutation of Cys45 to serine (C45S) disrupts the nucleophilic cysteine essential for catalysis, whereas simultaneous substitution of Cys45 and His148 (C45S/H148A) abolishes both catalytic elements while retaining protein stability.

Co-immunoprecipitation assays showed that both OTUD4 catalytic mutants retained the ability to physically associate with ZMYND8 at levels comparable to wild-type OTUD4 (Fig. 8C), indicating that loss of catalytic activity does not impair OTUD4–ZMYND8 binding. However, ubiquitination assays demonstrated that neither C45S nor C45S/H148A was able to remove ubiquitin chains from ZMYND8 (Fig. 8D), in contrast to the clear deubiquitination observed with wild-type OTUD4. These findings establish that OTUD4 regulates ZMYND8 turnover in a catalysis-dependent manner, further confirming that OTUD4 functions as the bona fide deubiquitinase responsible for ZMYND8 stabilization.

### OTUD4 deubiquitinates ZMYND8 at lysine 294 (human) / 318 (mouse)

**Fig. 9:**
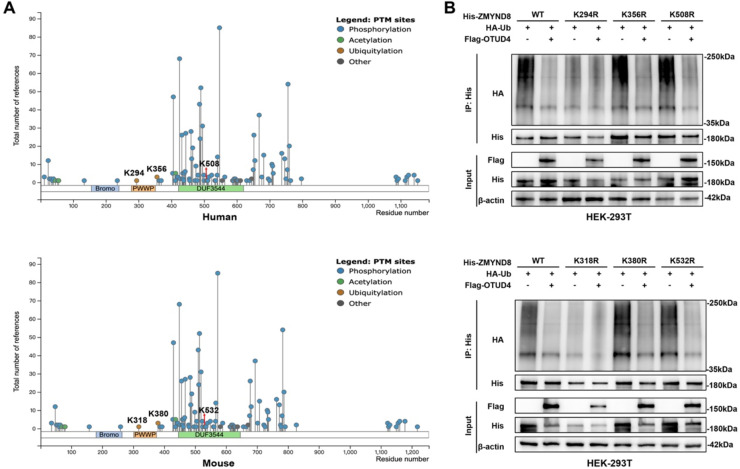
OTUD4 Deubiquitinates ZMYND8 at the K294 (Human)/K318 (Mouse) Sites. (A) Putative ubiquitination sites on ZMYND8 protein. (B) HEK-293T cells were co-transfected with plasmids expressing His-ZMYND8 (wild-type or mutant), HA-Ub, and Flag-OTUD4, treated with 10 μM MG132 for 8 hours before harvesting, and subjected to immunoprecipitation with an anti-His antibody followed by immunoblotting with an anti-HA antibody to detect ZMYND8 ubiquitination levels. Data shown are representative of three independent experiments.

To further identify lysine residues that may be critical for OTUD4-mediated deubiquitination of ZMYND8, we utilized the PhosphositePlus database (https://www.phosphosite.org/homeAction) to predict potential ubiquitination sites on ZMYND8. The analysis indicated that lysine residues K294, K356, and K508 are potential ubiquitination sites in human ZMYND8, while K318, K380, and K532 are corresponding candidate sites in mouse ZMYND8 (Fig. 9A). We then constructed point mutation plasmids in which lysine (Lys) was mutated to arginine (Arg), which cannot be ubiquitinated. These mutants were co-transfected with Flag-OTUD4 and HA-Ub into HEK-293T cells, followed by co-immunoprecipitation (Co-IP) assays. The results revealed that among all ubiquitination site mutants, only the K294R (human) and K318R (mouse) mutants exhibited significantly reduced ubiquitination levels (Fig. 9B). Moreover, when co-transfected with Flag-OTUD4, overexpression of OTUD4 decreased ubiquitination of ZMYND8 wild-type (WT; human), K356R (human), K508R (human), WT (mouse), K380R (mouse), and K532R (mouse) mutants, but failed to reduce ubiquitination of the ZMYND8 K294R (human) and K318R (mouse) mutants (Fig. 9B). These findings indicate that lysine residues K294 in human ZMYND8 and K318 in mouse ZMYND8 are specific sites targeted by OTUD4 for deubiquitination. It is worth noting that the slightly elevated His-ZMYND8 levels observed in the Flag-OTUD4 input lanes reflect the stabilizing effect of OTUD4 on ZMYND8, consistent with our findings in Fig.s 7F–G, and do not influence the interpretation of ubiquitination levels obtained from the His immunoprecipitation.

## Discussion

**Fig. 10:**
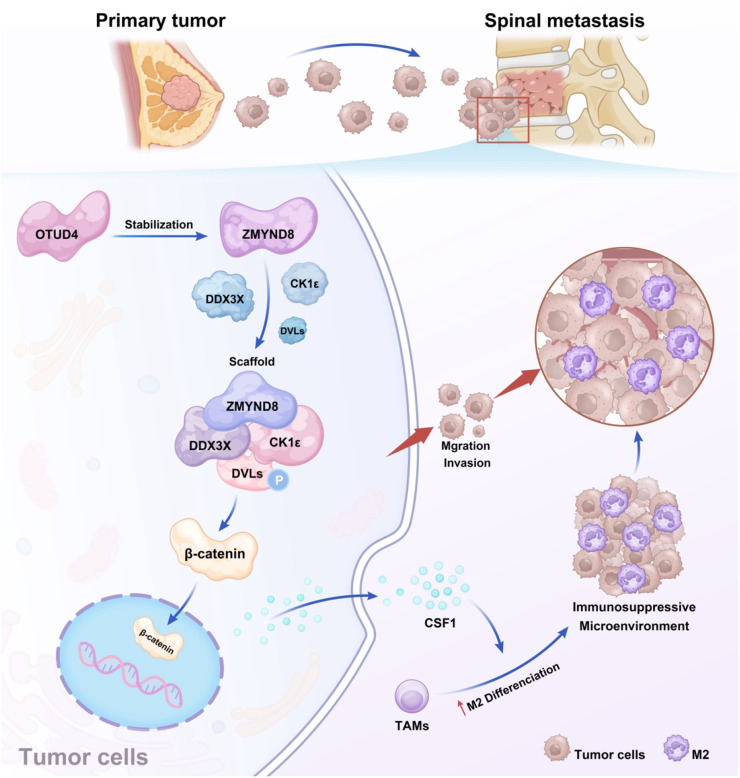
Schematic model illustrating the proposed role of the OTUD4-ZMYND8-DDX3X axis in spinal metastasis of TNBC. ZMYND8 is upregulated in spinal metastatic lesions and promotes TNBC dissemination through facilitating the assembly of the DDX3X-CK1ε complex, which activates WNT/β-catenin signaling. Furthermore, the deubiquitinase OTUD4 directly binds and stabilizes ZMYND8, thereby promoting its aberrant accumulation. The resulting OTUD4-ZMYND8-DDX3X axis drives canonical WNT/β-catenin signaling, upregulates CSF1 expression and promotes M2 polarization of macrophages, collectively fostering invasive behavior and establishing an immunosuppressive niche conducive to spinal metastasis.

Triple-negative breast cancer (TNBC) is characterized by a high propensity for spinal metastasis, a progression associated with substantial morbidity and poor survival outcomes [52,53]. The aggressive advancement of spinal lesions is closely linked to an immunosuppressive tumor microenvironment that supports tumor cell survival and facilitates immune evasion [54,55]. This study delineates a novel molecular pathway through which the deubiquitinating enzyme OTUD4 stabilizes the epigenetic reader protein ZMYND8, thereby promoting its functional interaction with the RNA helicase DDX3X. The resulting OTUD4–ZMYND8–DDX3X signaling axis drives canonical WNT/β-catenin signaling and upregulates CSF1 expression, collectively fostering invasive behavior and establishing an immunosuppressive niche conducive to spinal metastasis (Fig. 10).

The stabilization of ZMYND8 by OTUD4 introduces a previously unrecognized regulatory mechanism in TNBC progression. Although ZMYND8 has been implicated in transcriptional coactivation and epigenetic modulation across various cancers, the mechanisms governing its protein stability, particularly in metastatic contexts, have remained poorly understood. Recent work has identified USP7 as a deubiquitinase for ZMYND8 that promotes breast cancer cell migration and invasion[34], underscoring the importance of DUBs in modulating ZMYND8-driven oncogenic programs. In our TNBC and TNBC-SM models, however, unbiased ZMYND8 interactome profiling pointed to OTUD4 as the predominant ZMYND8-associated DUB. Our mechanistic investigations reveal that OTUD4 directly interacts with ZMYND8 and catalyzes its deubiquitination at evolutionarily conserved residues (K294 in human, K318 in murine) within the PWWP domain. This specific post-translational modification substantially enhances ZMYND8 protein stability and prolongs its half-life. Consequently, stabilized ZMYND8 facilitates the assembly of a transcriptional complex with DDX3X and CK1ε, culminating in DVL phosphorylation and sustained activation of the WNT/β-catenin pathway—a well-established regulator of epithelial-mesenchymal transition (EMT) and cellular invasion [46,56,57]. Furthermore, ZMYND8 upregulation augments the secretion of CSF1, a pivotal chemokine instrumental in recruiting and polarizing tumor-associated macrophages (TAMs) toward an M2-like [58], immunosuppressive phenotype that supports metastatic progression (Fig. 4, Fig. 5, Fig. 6). Consistent with prior reports indicating that ZMYND8 suppresses the cGAS–STING pathway and inhibits T-cell infiltration [29], our data suggest that OTUD4-mediated enhancement of ZMYND8 stability may amplify these immunoevasive programs, although additional *in vivo* validation is necessary to fully elucidate this link within the spinal metastatic niche.

These findings corroborate and extend previous reports implicating ZMYND8 in breast cancer pathogenesis through diverse mechanisms, including metabolic reprogramming [30], enhancement of antioxidant defenses via NRF2 [59], and suppression of the cGAS–STING pathway [29]. The identification of DDX3X as a functional binding partner provides a mechanistic foundation for ZMYND8-mediated hyperactivation of WNT signaling, particularly within the osseous niche where aberrant WNT activation is recognized to potentiate osteolytic destruction and immune suppression [[60], [61], [62]]. Similarly, OTUD4 has previously been demonstrated to promote metastatic dissemination by stabilizing oncoproteins such as Snail1 [38]. Our investigation expands this paradigm by identifying ZMYND8 as a novel substrate of OTUD4, highlighting a coordinated post-translational regulatory axis that amplifies pro-metastatic signaling.

Clinically, elevated ZMYND8 expression is associated with advanced tumor stage, lymph node metastasis, spinal involvement, and diminished survival in TNBC patients. The unique microenvironment of the spinal metastatic niche, characterized by hypoxia, acidic pH, and elevated calcium ion concentrations, may further potentiate ZMYND8 expression and functionality through pathways involving hypoxia-inducible factors and mechanosensitive signaling cascades such as YAP/TAZ, suggesting a self-reinforcing cycle that sustains metastatic signaling [63].

From a therapeutic perspective, targeting the OTUD4–ZMYND8–DDX3X axis represents a promising strategic approach for the management of TNBC-SM. Inhibitors designed to disrupt the OTUD4–ZMYND8 interaction or impede DDX3X–CK1ε complex formation may attenuate WNT pathway activation and reverse local immunosuppression. Combination therapeutic regimens incorporating immune checkpoint inhibitors hold potential to restore antitumor immunity and improve clinical outcomes.

While this study provides substantive mechanistic insight into the role of the OTUD4–ZMYND8–DDX3X axis, additional investigations are necessary to fully elucidate its chromatin-level regulatory mechanisms and *in vivo* functions within immunocompetent model systems. Subsequent multi-center clinical validation studies will also be essential to comprehensively assess its prognostic utility across molecular subtypes of TNBC.

In summary, we have characterized a novel OTUD4–ZMYND8–DDX3X signaling axis that promotes spinal metastasis in TNBC through concerted activation of WNT pathway signaling and formation of an immunosuppressive niche. These findings significantly advance our understanding of the molecular basis of TNBC metastasis and provide a compelling rationale for therapeutic targeting of this pathway.

## Abbreviations

**Table:** 

TNBC	Triple-negative breast cancer
TNBC-SM	Triple-negative breast cancer spinal metastasis
BRCA	Breast cancer
ZMYND8	Zinc finger MYND-type containing 8
OTUD4	OTU domain-containing protein 4
DDX3X	DEAD-box helicase 3 X-linked
DVL	Dishevelled family proteins
CK1ε	Casein kinase 1 epsilon
CSF1	Colony stimulating factor 1
DUB	Deubiquitinating enzyme
EMT	Epithelial–mesenchymal transition
TME	Tumor microenvironment
TAM	Tumor-associated macrophage
iBMDM	Immortalized bone marrow-derived macrophage
LC–MS/MS	Liquid chromatography–tandem mass spectrometry
IP–MS	Immunoprecipitation-Mass Spectrometry
Co-IP	Co-immunoprecipitation
PLA	Proximity ligation assay
IHC	Immunohistochemical
BLI	Bioluminescence imaging
q-PCR	Quantitative real time polymerase chain reaction
BCA	Bicinchoninic acid
PMA	Phorbol-12-myristate-13-acetate
OE	Overexpressing
Lys	Lysine
Arg	Arginine
STR	Short tandem repeat
CDSs	Coding sequences
cDNA	Complementary DNA
TCGA	The Cancer Genome Atlas
OS	Overall survival
DFS	Disease-free survival

## Ethics approval and consent to participate

All animal experiments were conducted in accordance with protocols approved by the Animal Care and Use Committee of Zhongshan Hospital, Fudan University (Shanghai, China) (Approval No 2024-038). The use of clinical specimens was authorized by the Medical Ethics Committee of Zhongshan Hospital, Fudan University (Shanghai, China) (Approval No B2023-296).

**Data availability**

All data supporting this paper are presented within the paper and/or the Supplementary Materials. The original data sets are also available from the corresponding author upon reasonable request.

## Funding

This work was supported by the National Natural Science Foundation of China (Grant Nos. 82172738, 82473356).

**Consent for publication**

Not applicable.

**Declaration of generative AI use**

Generative AI and AI-assisted technologies were NOT used in the preparation of this work.

## CRediT authorship contribution statement

**Bing Liang:** Writing – original draft, Visualization, Validation, Software, Methodology, Investigation, Formal analysis, Data curation, Conceptualization. **Annan Hu:** Visualization, Validation, Methodology, Investigation. **Hongwei Lu:** Visualization, Validation, Software, Methodology. **Hao Zhou:** Validation, Software, Methodology, Data curation. **Qing Chen:** Validation, Software, Methodology. **Chao Jia:** Validation, Methodology, Investigation. **Jinjin Wang:** Software, Methodology. **Libo Jiang:** Writing – review & editing, Project administration, Methodology, Investigation, Conceptualization. **Wei Hong:** Writing – review & editing, Methodology. **Jian Zhou:** Writing – review & editing, Methodology. **Jian Dong:** Writing – review & editing, Validation, Supervision, Project administration, Investigation, Funding acquisition, Conceptualization.

## Declaration of competing interest

The authors declare that they have no known competing financial interests or personal relationships that could have appeared to influence the work reported in this paper.

## Appendix Supplementary materials

Image, application 1
