# Supplementary material for: OTUD4-ZMYND8-DDX3X Axis Drives Immunosuppressive Microenvironment in Spinal Metastases of Triple-Negative Breast Cancer
Source: Neoplasia. 2025 Nov 25;71:101259. doi: 10.1016/j.neo.2025.101259 (PMC12686931; doi:10.1016/j.neo.2025.101259)
Supplement: Supplementary file 1 [file mmc1.docx]

**OTUD4-ZMYND8-DDX3X Axis Drives Immunosuppressive Microenvironment in Spinal Metastases of Triple-Negative Breast Cancer**

Bing Liang^1, 2†^, Annan Hu^1†^, Hongwei Lu^1†^, Hao Zhou^3†^, Qing Chen^1^, Chao Jia^1^, Jinjin Wang^1^, Libo Jiang^1, 5^ *, Wei Hong^4^ *, Jian Zhou^1^ * and Jian Dong^1, 2, 5, 6^ *

^1^Department of Orthopaedic Surgery, Zhongshan Hospital, Fudan University, Shanghai 200032, China

^2^Department of Orthopaedic Surgery, Shanghai Geriatric Medical Center, Shanghai 201104, China

^3^Department of Orthopaedic Surgery, Shanghai Xuhui Central Hospital, Zhongshan-Xuhui Hospital, Fudan University, Shanghai 200031, China

^4^Department of Geriatrics and Gerontology, Huadong Hospital, Fudan University, Shanghai 200040, China

^5^State Key Laboratory of Molecular Engineering of Polymers, Fudan University, Shanghai 200438, China

^6^Department of Orthopaedic Surgery Zhongshan Hospital Wusong Branch Fudan University, Shanghai 200940, China

† Bing Liang, Annan Hu, Hongwei Lu and Hao Zhou contributed equally to this work.

* Corresponding author:

Libo Jiang (jiang.libo@zs-hospital.sh.cn), Wei Hong (drivyh@126.com), Jian Zhou (zhou.jian1@zs-hospital.sh.cn), Jian Dong (dong.jian@zs-hospital.sh.cn)

**This file contains Supplementary Figures S1–S7 and Tables S1–S2.
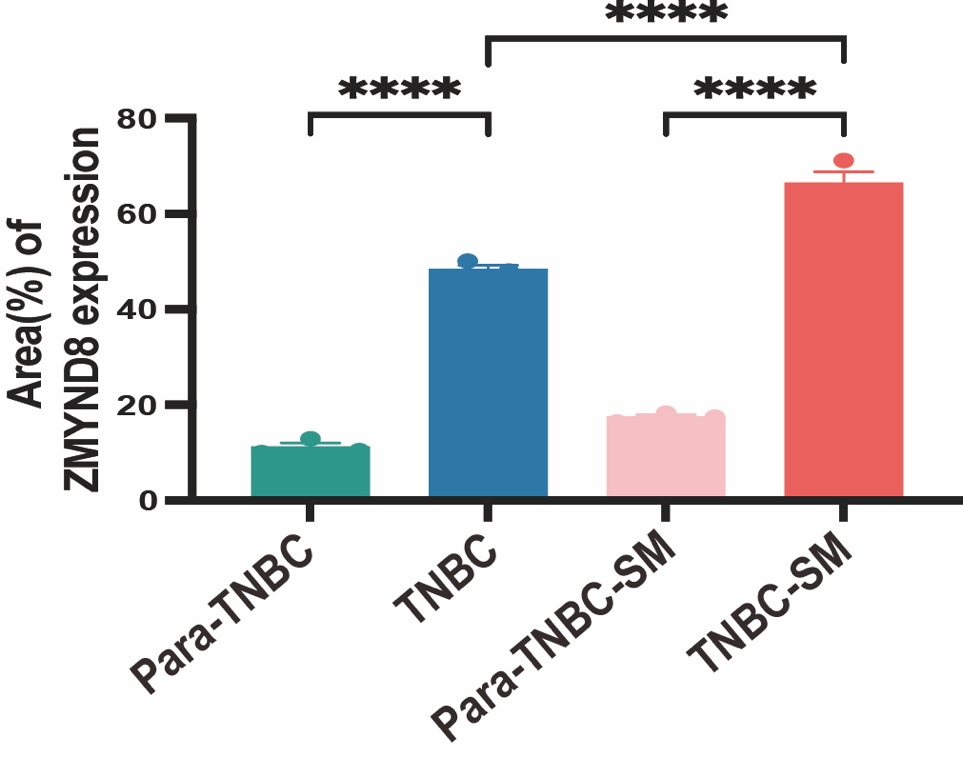
**

**Figure S1.** Immunohistochemical (IHC) Analysis of ZMYND8 Expression in TNBC, TNBC Spinal Metastasis (TNBC-SM), and Corresponding Adjacent Normal Tissues. Representative IHC images are shown along with quantitative analysis of ZMYND8-positive staining areas. Data in (S1) are presented as mean ± SD and analyzed by one-way ANOVA. *p < 0.05, **p < 0.01, ***p < 0.001, ****p < 0.0001.


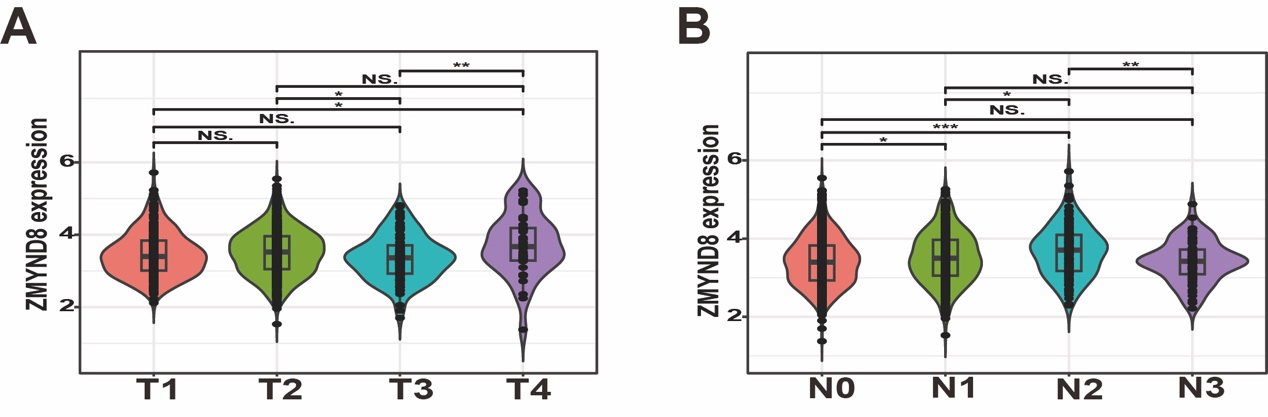


**Figure S2.** (A) Expression levels of ZMYND8 across different tumor stages (T1–T4) of breast cancer based on the TCGA dataset; (B) ZMYND8 expression stratified by lymph node metastasis status (N0–N3) in breast cancer based on the TCGA dataset.


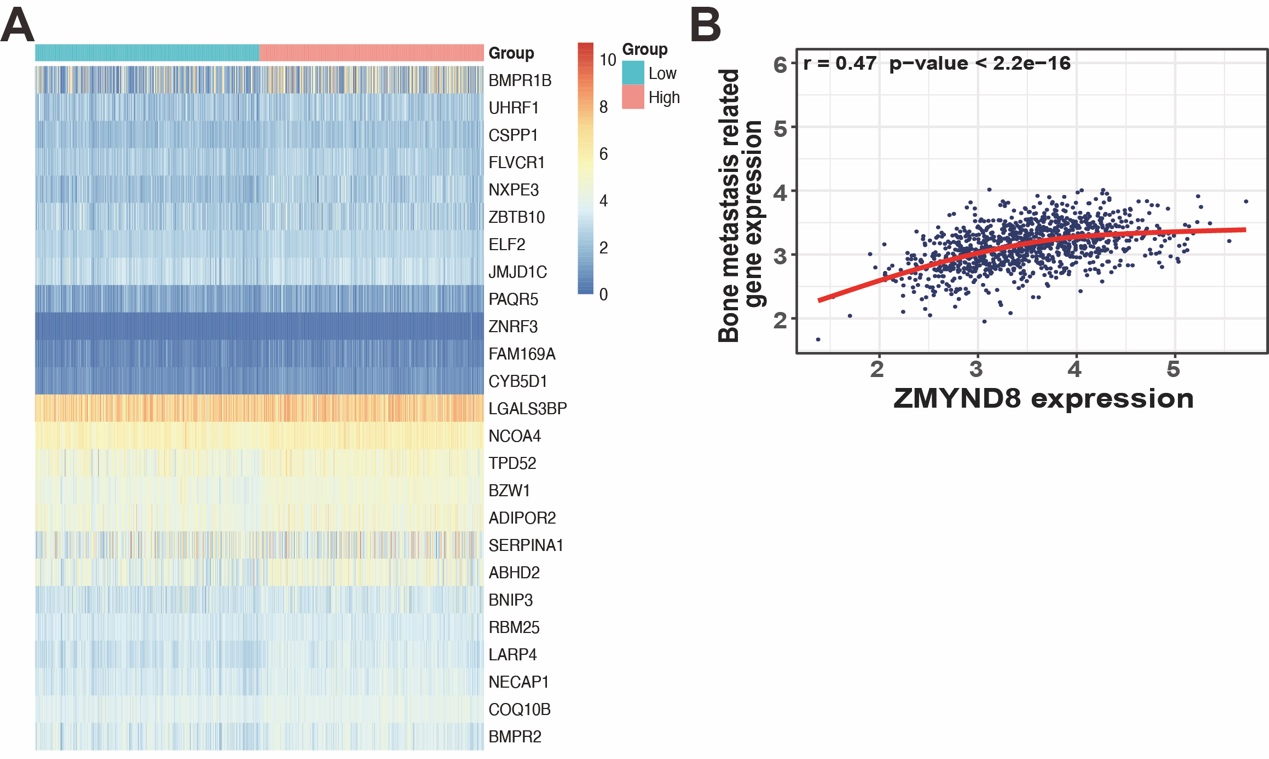


**Figure S3.** ZMYND8 Is Upregulated in Breast Cancer and Associated with Bone Metastasis. Bone metastasis-associated genes were screened from the MSigDB database and analyzed for correlation. (A) Heatmap showing genes differentially expressed in correlation with ZMYND8. (B) Correlation analysis between ZMYND8 expression levels and bone metastasis-associated genes.


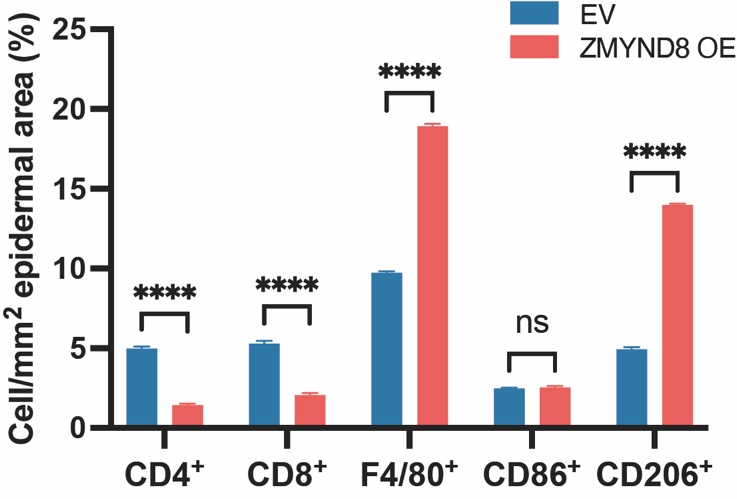


**Figure S4.** Quantitative analysis of CD4⁺, CD8⁺, F4/80⁺, CD86⁺, and CD206⁺ positive staining areas in 4T1 mouse breast cancer tissues by immunohistochemistry. Data in (S4) are presented as mean ± SD and analyzed by one-way ANOVA. *p < 0.05, **p < 0.01, ***p < 0.001, ****p < 0.0001.


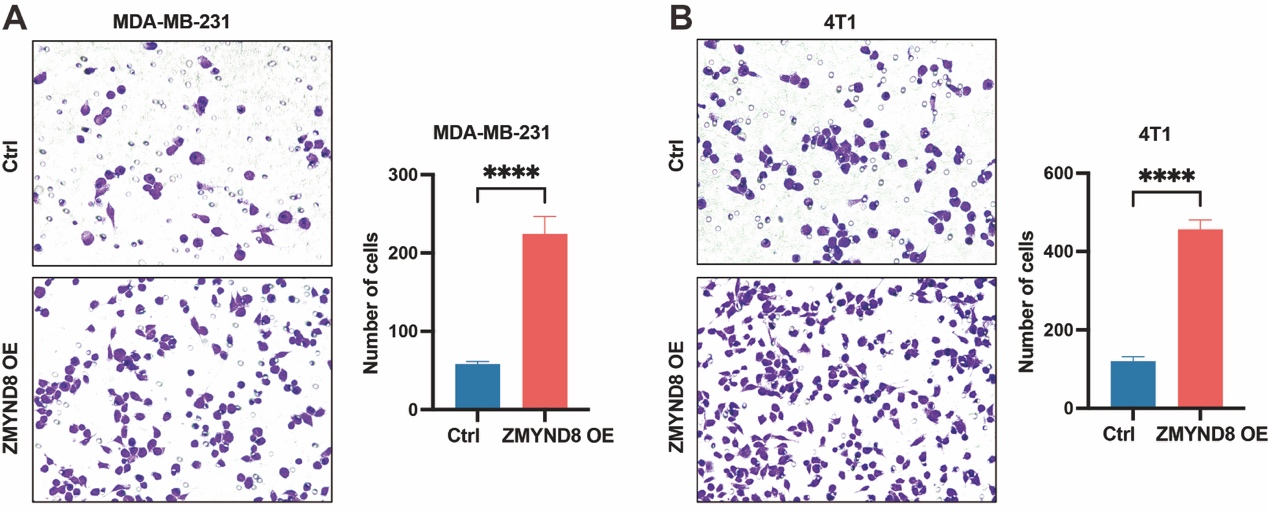


**Figure S5.** ZMYND8 Overexpression Enhances the Migratory Capacity of Tumor-Associated Macrophages in Breast Cancer. (A, B) Transwell migration assay evaluating macrophage migratory capacity following coculture with breast cancer cells expressing different levels of ZMYND8. Data in (S5) are presented as mean ± SD and analyzed by Student’s t-test. *, p < 0.05; **, p < 0.01; ***, p < 0.001; ****, p < 0.0001. Data shown are representative of three independent experiments.


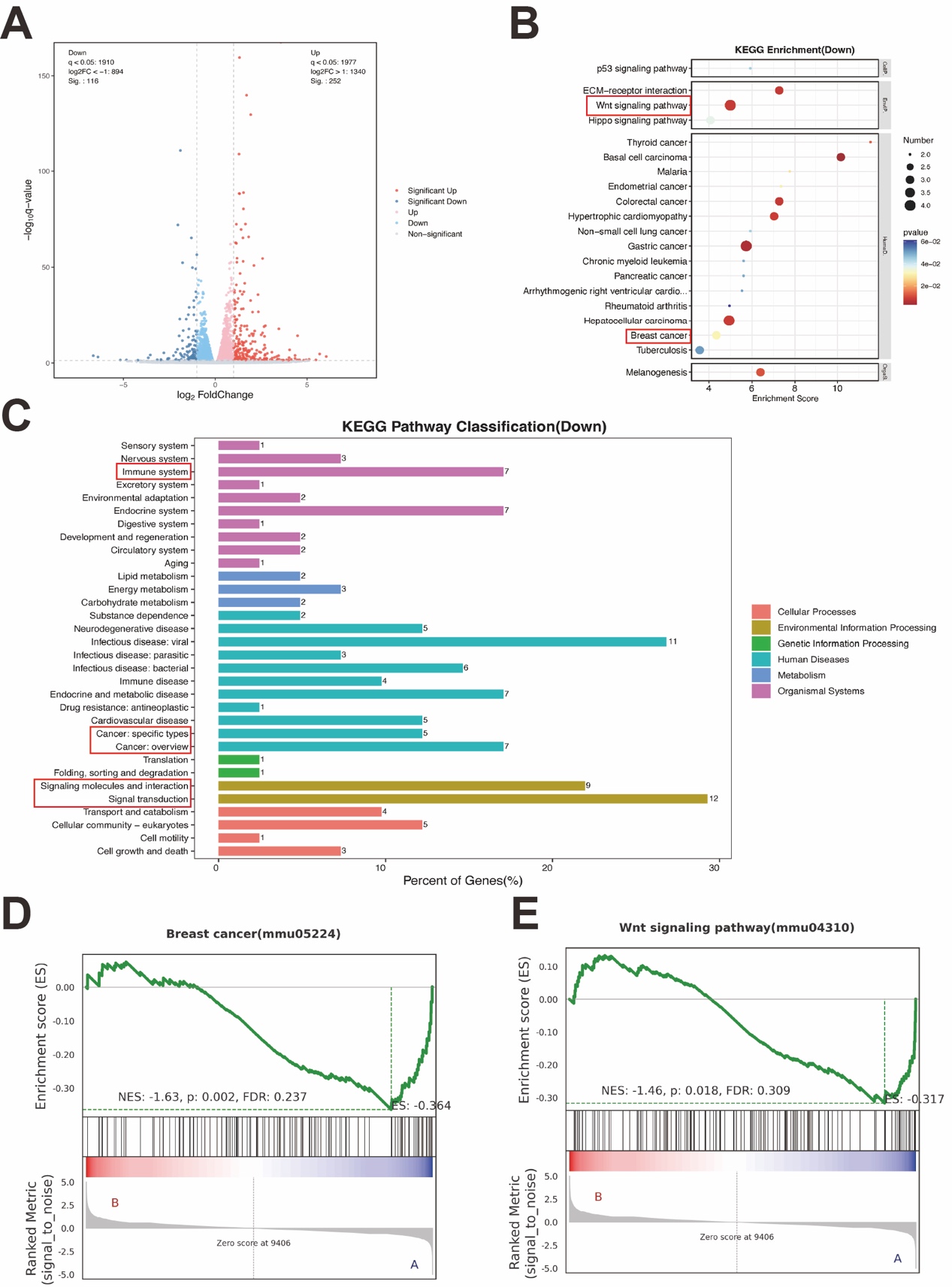


**Figure S6.** ZMYND8 Regulates the WNT Signaling Pathway in Breast Cancer. Transcriptome sequencing was performed in ZMYND8-knockout and control groups of MDA-MB-231 cells. (A) Volcano plot displaying differentially expressed genes from RNA-Seq (∣log₂FC∣ > 1, p < 0.05). (B, C) KEGG pathway analysis of the differentially expressed genes. (D, E) Gene Set Enrichment Analysis (GSEA) showing enrichment of differential genes in the breast cancer pathway and Wnt signaling pathway. The enrichment score (ES) is indicated.


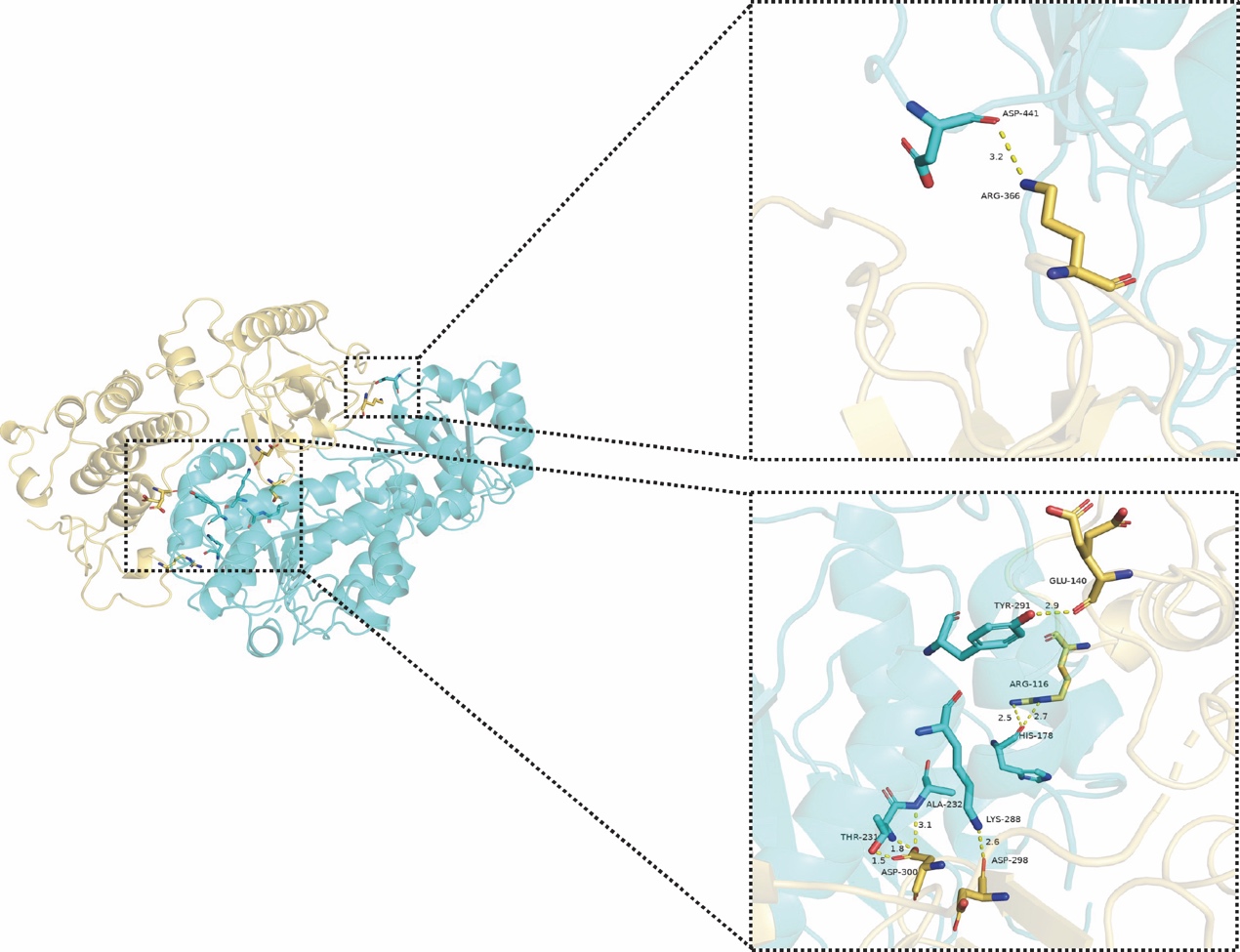


**Figure S7.** RosettaDock analysis predicted the interaction between ZMYND8 and DDX3X.

**Table S1.** Sequence of qRT-PCR primers

| **Gene** | **Sequence (5' - 3')** |
| --- | --- |
| h*GAPDH* | Forward Primer: ACAACTTTGGTATCGTGGAAGG  Reverse Primer: GCCATCACGCCACAGTTTC |
| h*ZMYND8* | Forward Primer: GGGTTTATCACGCTAAGTGTCTG  Reverse Primer: GGCTTTACTCTGGGTCTCGATG |
| h*CD206* | Forward Primer: TCCGGGTGCTGTTCTCCTA  Reverse Primer: CCAGTCTGTTTTTGATGGCACT |
| m*CD206* | Forward Primer: CTCTGTTCAGCTATTGGACGC  Reverse Primer: TGGCACTCCCAAACATAATTTGA |
| h*ARG1* | Forward Primer: GTGGAAACTTGCATGGACAAC  Reverse Primer: AATCCTGGCACATCGGGAATC |
| m*ARG1* | Forward Primer: CTCCAAGCCAAAGTCCTTAGAG  Reverse Primer: GGAGCTGTCATTAGGGACATCA |
| h*IL-10* | Forward Primer: GACTTTAAGGGTTACCTGGGTTG  Reverse Primer: TCACATGCGCCTTGATGTCTG |
| m*IL-10* | Forward Primer: CTTACTGACTGGCATGAGGATCA  Reverse Primer: GCAGCTCTAGGAGCATGTGG |
| h*TGF-β* | Forward Primer: GGCCAGATCCTGTCCAAGC  Reverse Primer: GTGGGTTTCCACCATTAGCAC |
| m*TGF-β* | Forward Primer: CCACCTGCAAGACCATCGAC  Reverse Primer: CTGGCGAGCCTTAGTTTGGAC |

**Table S2.** Information of antibodies used in the current study

| **Name** | **Vendor** | **Catalog number** |
| --- | --- | --- |
| ZMYND8 Polyclonal antibody | Proteintech | 11633-1-AP |
| ZMYND8 monoclonal antibody (M01) | Abnova | H00023613-M01 |
| Beta Actin Polyclonal antibody | Proteintech | 20536-1-AP |
| DDX3 Polyclonal antibody | Proteintech | 11115-1-AP |
| DVL2 Polyclonal antibody | Proteintech | 12037-1-AP |
| OTUD4 Polyclonal antibody | Proteintech | 25070-1-AP |
| Anti-Dishevelled 2 (phospho S143) | Abcam | ab124933 |
| CSNK1E Rabbit pAb | ABclonal | A1796 |
| β-Catenin Rabbit mAb | ABclonal | A19657 |
| CSF1 Rabbit pAb | ABclonal | A1627 |
| DDDDK-Tag Rabbit mAb | ABclonal | AE092 |
| Rabbit anti His-tag mAb | ABclonal | AE086 |
| HA-Tag Rabbit mAb | ABclonal | AE105 |
| HRP-conjugated Goat Anti-Rabbit IgG (H+L) | Proteintech | SA00001-2 |
